# Huayu Qutan Recipe promotes lipophagy and cholesterol efflux through the mTORC1/TFEB/ABCA1‐SCARB1 signal axis

**DOI:** 10.1111/jcmm.18257

**Published:** 2024-03-25

**Authors:** Yue Li, Jiaxiang Pan, J. J. Jiajia Yu, Xize Wu, Guanlin Yang, Xue Pan, Guoyuan Sui, Mingyang Wang, Meijia Cheng, Shu Zhu, He Tai, Honghe Xiao, Lili Xu, Jin Wu, Yongju Yang, Jing Tang, Lihong Gong, Lianqun Jia, Dongyu Min

**Affiliations:** ^1^ Department of Cardiology the Affiliated Hospital of Liaoning University of Traditional Chinese Medicine Shenyang China; ^2^ Liaoning Provincial Key Laboratory of TCM Geriatric Cardio‐Cerebrovascular Diseases Shenyang China; ^3^ Graduate School of Liaoning University of Traditional Chinese Medicine Shenyang China; ^4^ Postdoctoral Program of Liaoning University of Traditional Chinese Medicine Shenyang China; ^5^ Nantong Hospital of Traditional Chinese Medicine Nantong Hospital Affiliated to Nanjing University of Chinese Medicine Nantong China; ^6^ Innovation Engineering Technology Center of Traditional Chinese Medicine Liaoning University of Traditional Chinese Medicine Shenyang China; ^7^ Dazhou Vocational College of Chinese Medicine Dazhou China; ^8^ College of Animal Science and Veterinary Medicine of Shenyang Agricultural University Shenyang China; ^9^ Experimental Center of Traditional Chinese Medicine the Affiliated Hospital of Liaoning University of Traditional Chinese Medicine Shenyang China; ^10^ Department of Paediatric Dentistry, School of Stomatology China Medical University Shenyang China; ^11^ School of Pharmacy Liaoning University of Traditional Chinese Medicine Dalian China; ^12^ Department of Cardiology, 924 Hospital of Joint Logistic Support Force of PLA Guilin China

**Keywords:** autophagy, lipid metabolism, macrophage, ox‐LDL

## Abstract

This study aims to investigate the mechanism of the anti‐atherosclerosis effect of Huayu Qutan Recipe (HYQT) on the inhibition of foam cell formation. In vivo, the mice were randomly divided into three groups: CTRL group, MOD group and HYQT group. The HYQT group received HYQT oral administration twice a day (20.54 g/kg/d), and the plaque formation in ApoE^−/−^ mice was observed using haematoxylin–eosin (HE) staining and oil red O (ORO) staining. The co‐localization of aortic macrophages and lipid droplets (LDs) was examined using fluorescent labelling of CD11b and BODIPY fluorescence probe. In vitro, RAW 264.7 cells were exposed to 50 μg/mL ox‐LDL for 48 h and then treated with HYQT for 24 h. The accumulation of LDs was evaluated using ORO and BODIPY. Cell viability was assessed using the CCK‐8 assay. The co‐localization of LC3b and BODIPY was detected via immunofluorescence and fluorescence probe. LysoTracker Red and BODIPY 493/503 were used as markers for lysosomes and LDs, respectively. Autophagosome formation were observed via transmission electron microscopy. The levels of LC3A/B II/LC3A/B I, p‐mTOR/mTOR, p‐4EBP1/4EBP1, p‐P70S6K/P70S6K and TFEB protein level were examined via western blotting, while SQSTM1/p62, Beclin1, ABCA1, ABCG1 and SCARB1 were examined via qRT‐PCR and western blotting. The nuclear translocation of TFEB was detected using immunofluorescence. The components of HYQT medicated serum were determined using Q‐Orbitrap high‐resolution MS analysis. Molecular docking was employed to identify the components of HYQT medicated serum responsible for the mTOR signalling pathway. The mechanism of taurine was illustrated. HYQT has a remarkable effect on atherosclerotic plaque formation and blood lipid level in ApoE^−/−^ mice. HYQT decreased the co‐localization of CD11b and BODIPY. HYQT (10% medicated serum) reduced the LDs accumulation in RAW 264.7 cells. HYQT and RAPA (rapamycin, a mTOR inhibitor) could promote cholesterol efflux, while chloroquine (CQ, an autophagy inhibitor) weakened the effect of HYQT. Moreover, MHY1485 (a mTOR agonist) also mitigated the effects of HYQT by reduced cholesterol efflux. qRT‐PCR and WB results suggested that HYQT improved the expression of the proteins ABCA1, ABCG1 and SCARB1.HYQT regulates ABCA1 and SCARB1 protein depending on the mTORC1/TFEB signalling pathway. However, the activation of ABCG1 does not depend on this pathway. Q‐Orbitrap high‐resolution MS analysis results demonstrated that seven core compounds have good binding ability to the mTOR protein. Taurine may play an important role in the mechanism regulation. HYQT may reduce cardiovascular risk by promoting cholesterol efflux and degrading macrophage‐derived foam cell formation. It has been observed that HYQT and ox‐LDL regulate lipophagy through the mTOR/TFEB signalling pathway, rather than the mTOR/4EBP1/P70S6K pathway. Additionally, HYQT is found to regulate cholesterol efflux through the mTORC1/TFEB/ABCA1‐SCARB1 signal axis, while taurine plays a significant role in lipophagy.

## 1 INTRODUCTION

Atherosclerosis (AS) is an arterial disease formed by the synergistic effect of a variety of pathological factors. The cardiovascular diseases (CVDs) fact sheet of the World Health Organization reported that more than 17 million people died from CVDs in 2015, representing 31% of all global deaths.^1^ Of these, an estimated 7.4 million occurred due to coronary heart disease and 6.7 million dueto stroke.^2^ Atherosclerotic cardiovascular disease remains a leading cause of vascular disease worldwide.

As is a leading cause of cardiovascular disease. From a pathogenesis perspective, atherosclerosis is a highly intricate disease involving factors like oxidative stress and endothelial damage. Inflammation and alterations in lipid metabolism are considered to be the primary components of atherogenesis.^3^ This study aims to specifically investigate lipid metabolism. Statins, as the most effective anti‐atherosclerosis drugs, have many problems in their long history of clinical use. More than 50% of patients receiving intensive statin therapy experienced side effects such as diabetes, muscular disease, hepatotoxicity and nephrotoxicity.^4^ Due to the complex aetiology and pathogenesis of AS, it is difficult to achieve the ideal therapeutic effect with single‐target drugs. At present, Traditional Chinese Medicine (TCM) has achieved good results in the prevention and treatment of atherosclerotic diseases.

Lipophagy, a specialized form of autophagy, is responsible for the selective degradation of lipid droplets (LDs) and plays a crucial role in maintaining cellular lipid homeostasis. Lipophagy, a process in which lipid droplets are degraded into free cholesterol, has emerged as a novel strategy for treating AS.^5^ MTOR is a crucial gene involved in regulating the lipophagy signal. It exhibits sensitivity to various nutrients, such as amino acids and glucose. Once the lysosomal autophagy pathway is triggered due to starvation, mTOR plays a significant role in lipid catabolic metabolism. Transcription factor EB (TFEB), eIF4E‐binding protein (4EBP1) and p70 Ribosomal Protein S6 Kinase (P70S6K) are downstream molecules of mTOR.

TFEB is a member of the MiTF/TFE family of transcription factors, gets phosphorylated by mTORC1 when nutrient‐rich conditions prevail. This phosphorylation causes TFEB to bind to its cytoplasmic chaperone, YWHA/14–3‐3 and remain in the cytoplasm.^6^, ^7^ Inhibition of mTOR results in the de‐phosphorylation of TFEB, leading to its translocation into the nucleus.^8^, ^9^, ^10^ When TFEB is defective, it leads to the accumulation of lipid droplets within the cells. As a result, TFEB is recognized as a critical molecule in the treatment of metabolic diseases. Additionally, this signalling pathway plays a crucial role in regulating the cholesterol content in macrophages and promoting cholesterol efflux through lipid droplet autophagy.^11^ MTOR/4EBP1/P70S6K is an important pathway of lipid metabolic. 4EBP1 and p70S6K are direct substrates of mTOR, and play an important role in protein translation. After mTOR activation, the downstream effectors 4EBP1 and P70S6K are phosphorylated to promote the anabolic process.

Additionally, regarding strategies for reducing cholesterol accumulation, the main approaches involve inhibiting intake and promoting efflux. In oxLDL‐loaded macrophages, cholesterol can be transported outside the cell through transport proteins like ABCA1, ABCG1 and SCARB1. This cholesterol efflux by macrophages plays a crucial role in reducing foam cell formation, making it an important pathway for mitigating AS.^12^, ^13^, ^14^

According to TCM, the sensation of ‘chest discomfort’ can be attributed to ‘spleen deficiency’ and the presence of ‘phlegm and stasis’, which ultimately results in ‘blood vessel obstruction’. However, there is currently no research available on the impact of TCM in terms of invigorating qi, invigorating the spleen, removing stasis and removing phlegm on macrophage lipophagy. HYQT, a modified formula that integrates Chinese and Western medical theories, has been found to possess anti‐hyperlipidaemic^15^ and atherosclerotic effects in previous studies. Moreover, HYQT plays a significant role in the regulation of autophagy.^16^, ^17^, ^18^, ^19^

Based on these findings, it has been hypothesized that HYQT may have a regulatory effect on autophagy and cholesterol efflux. This study aimed to investigate the mechanism of the mTOR pathway in oxLDL‐induced foam cell formation. Additionally, it aimed to explore the potential significance of HYQT in the context of anti‐atherosclerosis by examining its role in regulating the mTOR signalling pathway.

## 2 MATERIALS AND METHODS

### 2.1 Materials

The following reagents were used: haematoxylin—and eosin (HE) staining kit (Servicebio, Cat.G1005); Oil red staining solution (Servicebio, Cat.G1016); Mounting Medium, antifading (with DAPI) (Solarbio, Lot.20210106); Total cholesterol (TC) (Nanjing Jiancheng Technology, CAT. A111‐1‐1), triglycerides (TG) (Nanjing Jiancheng Technology, CAT.A110‐1‐1); High‐density lipoprotein cholesterol (HDL‐C) (Nanjing Jiancheng Technology, CAT.A112‐1‐1); Low‐density lipoprotein cholesterol level (LDL‐C) (Nanjing Jiancheng Technology, CAT. A113‐1‐1); Cell count kit‐8 (CCK 8) (Apexbio, Cat. K10185133EF5E); Paraformaldehyde, 4% (Solarbio, Lot. 20180130); CQ (Solarbio, Cat. SC5190); MHY1485 (MedCHemExpress, Cat. 64066); Rapamycin (Solarbio, Cat.1112P033; DMSO 25 mg/mL); Ox‐LDL (Yiyuan Biotech, Cat. YB‐002) and HDL (Solarbio, Cat. H7940); Lyso‐Tracker Red (Solarbio, Lot. 20201111); NBD‐cholesterol (Ruixibio, Cat. RJ0211907); BODIPY 493/503 (MaoKangbio, Lot. 1912X200515); CheKine Free Cholesterol (FC) Colorimetric Assay Kit (Abbkine, Cat. ATTOC3001); RIPA Lysis Buffer (Beyotime, Cat, P0013B); The Enhanced BCA Protein Assay Kit was adopted to determine protein concentrations (Beyotime, Cat. P0010S); Taurine (Macklin, Cat, 107‐35‐7).

The following primary antibodies were used: Anti‐CD11b (PTM Bio, Cat. PTM‐6088); Anti‐SQSTM1 (Abclonal, Cat.A19700); Anti‐Beclin1 (Cell Signaling Technology, Cat. 3495); Anti‐LC3b (NOVUSBIO, Cat. NB100‐2220); Anti‐LC3A/B (Cell Signaling Technology, Cat. 12741); Anti‐p‐mTOR‐S2448 (Cell Signaling Technology, Cat. 5536); Anti‐mTOR (Cell Signaling Technology, Cat. 2983); Anti‐TFEB (ABBKINE, Cat. ABP56578); Anti‐Phospho‐eIF4EBP1‐S65 Rabbit (Abclonal, Cat.AP1363); Anti‐4EBP1 (Proteintech, Cat. 60246‐1‐Ig); Anti‐P70S6K (Abcam, Cat. ab32529); Anti‐p‐P70S6K (Proteintech, Cat. 14485‐1‐AP); Anti‐ABCA1 (Abclonal, Cat. A7228); Anti‐ABCG1 (Bioss, Cat. bs‐23382R); Anti‐SCARB1 (Bioss, Cat. bs‐23977R).

The following secondary antibodies were used for western blot and immunofluorescence experiments: Alexa Fluor 594‐conjugated goat anti‐rabbit IgG (H + L) (Abclonal, Cat. AS039); β‐Actin Rabbit mAb (Abclonal, Cat. AC038).

### 2.2 Methods

#### 2.2.1 Preparation of drugs

Huayu Qutan Recipe, including nine herbs, Codonopsis Radix (Family: Campanulaceae, *Codonopsis pilosula* (Franch), Dangshen in Chinese), Astragali Radix (Family: Leguminous, *Astragalus membranaceus* (Fisch), Huangqi in Chinese), Gynostemma pentaphyllum (Thunb.) Makino (Family: Cucurbitaceae, *Herba Gynostemmatis Pentaphylli*, Jiaogulan in Chinese), Salviae miltiorrhizae Rasix et Rhizoma (Family: Lamiaceae, *Salvia miltiorrhiza* Bge, Danshen in Chinese), Poria (Family: Polyporaceae, *Poria cocos* (Schow.) Wolf, Fuling in Chinese), Pinelliae Rhizoma (Family: Araceae, *Pinellia ternata* (Thunb.) Breit, Banxia in Chinese), Acori Tatarinowii Rhizoma (Family: Araceae, *Acorus tatarinowii* Schott, Shichangpu in Chinese), *Chuanxiong Rhizoma* (Family: Umbelliferae, *Ligusticum chuanxiong* Hort., Chuanxiong in Chinese), Curcumae Radix (Family: Zingiberaceae, *Curcuma wenyujin* Y. H. Chen et C. Ling, Wenyujin in Chinese), the herbs were obtained from the Affiliated Hospital of Liaoning University of Traditional Chinese Medicine. The ratios among these herbs were as 5: 5: 3: 1: 2: 1: 1: 1: 1 (Table 1).

**TABLE 1:** Huayu Qutan Recipe ingredients.

English name	Chinese name and Latin name	Family name	Medical part	Dosage (g)
Codonopsis Radix	Dang‐shen (Codonopsis pilosula (Franch).)	Campanulaceae	Dried root	25
Astragali Radix	Huang‐qi (Astragalus membranaceus (Fisch))	Leguminous	Dried root	25
Gynostemma pentaphyllum (Thunb.) Makino	Jiao‐gu‐lan (Herba Gynostemmatis Pentaphylli)	Cucurbitaceae	whole herb	15
Salviae miltiorrhizae Rasix et Rhizoma	Dan‐shen (Salvia miltiorrhiza Bge)	Lamiaceae	Dried root and rhizome	5
Poria	Fu‐ling (*Poria cocos* (Schow.) Wolf)	Polyporaceae	Dried sclerotia	10
Pinelliae Rhizoma	Ban‐xia (Pinellia ternata (Thunb.) Breit)	Araceae	Dried rhizome	5
Acori Tatarinowii Rhizoma	Shi‐chang‐pu (Acorus tatarinowii Schott)	Araceae	Dried root	5
Chuanxiong Rhizoma	Chuan‐xiong (Ligusticum chuanxiong Hort.)	Umbelliferae	Dried root and rhizome	5
Curcumae Radix	Yu‐jin (*Curcuma wenyujin* Y. H. Chen et C. Ling)	Zingiberaceae	Dried root	5

#### 2.2.2 Experimental animals and groups

A total of 12 C57BL/6J WT (wild type) mice and 24 of ApoE^−/−^ mice on a C57BL/6J background were purchased from Jiangsu Jicui Yaokang Biotechnology Co., Ltd. And Beijing Weitonglihua Laboratory Animal Technology Co., Ltd. Permit number: SCXK (JS) 2019‐0009 and SCXK (BJ) 2016‐0006. Before being exposed to different treatment, mice were adapt to the new experimental environment (at a temperature of 22 ± 1°C and a 12 h light–dark cycle, with a humidity of 45%–55%) for 1 week. ApoE^−/−^ mice were randomly divided into two groups, mice were fed with high‐fat diet for 16 weeks to induced atherosclerosis model. C57BL/6J (WT) were used for control group (CTRL), and ApoE^−/−^ mice were used for MOD group and HYQT group. Ethics approval by Animal Ethics Committee of Changchun Wish Testing Technology Service Co. (Ethics number: 20200612‐01).

The groups are as follows:① CTRL group (*n* = 12): WT mice were fed with normal diet for 16 weeks, received 0.9% normal saline by intragastric gavage for 8 weeks; ② MOD group (*n* = 12): ApoE^−/−^ mice were fed with HFD for 16 weeks, received 0.9% normal saline by intragastric gavage for 8 weeks (16.7 mL/kg/d); ③HYQT (human equivalent dose) group (*n* = 12): ApoE^−/−^ mice were fed with HFD for 16 weeks, received by the Huayu Qutan Recipe intragastric gavage for 8 weeks (20.54 g/kg/d). The dose of HYQT group based on the clinically recommended dose of 60 kg adult equivalent dose (HBE, human equivalent dose), and converted into the mice gavage dose according to the equivalent dose for mice and humans was 12.3 times (Table 2).High‐fat feed composed of lard, cholesterol and basic feed. The proportions of feed ingredients are as follows: lard 15%, cholesterol 0.25% and basic feed 84.75%.

**TABLE 2:** Conversion of animal doses to HED based on BSA.

Species	Weight(kg)	BSA(m^2^)	*K*_*m*_ factor
Human adult	60	1.6	37
Mouse	0.02	0.007	3
Rat	0.15	0.025	6

*Note*: HED (mg/kg) = animal dose (mg/kg) multiplied by (animal *K*_*m*_/human *K*_*m*_).

#### 2.2.3 Sample collection

After 16 weeks, 1% pentobarbital sodium (50 mg/kg) intraperitoneal injection was used to anaesthetise the mice. Measured the body weight, collected blood sample from mice orbit and centrifuged at 3000 rpm. Collected serum and stored it in a refrigerator at −80°C for subsequent experiments. The aortas of the mice were separated and stored in 4% paraformaldehyde and electron microscope fixator. −80°C refrigerator stored for oil red O staining and immunofluorescence staining.

#### 2.2.4 Lipid measurement

Blood from fasting mice was collected into EP tubs by orbital puncture. Centrifuged at 12000 rpm for 10 min at 4°C to collect the plasma. Detected total cholesterol (TC), triglycerides (TG), high‐density lipoprotein cholesterol (HDL‐C) and low‐density lipoprotein cholesterol level (LDL‐C), according to the manufacturer's protocols.

#### 2.2.5 Haematoxylin and eosin staining

The aortic roots were excised and fixed in 10% neutral formalin, then washed with various increasing concentrations of ethanol, decolorized, removed and then embedded in paraplast. The 4‐μm‐thick sections were stained with HE stain solution and observed with an optical microscope (Nikon Eclipse E100) to assess the degree of histopathological damage.

#### 2.2.6 Oil red O staining

Evaluated the plaque formation and lipid deposition of the aorta via oil red O staining. The aortas were processed according to the method of Bigford.^20^

#### 2.2.7 Co‐localization of CD11b and BODIPY

The immunofluorescence method was used to detect the macrophages stained by the specific antibody CD11b and the cholesterol ester fluorescent probe body to observe the co‐staining of macrophages and lipids in the plaque. In short, the CD11b primary antibody was treated overnight at 4°C after the BODIPY 493/503 incubated for 1 hour at room temperature away from light. Wash samples with PBS and conjugate them with the corresponding fluorescent secondary antibody. Then, on Mounting Medium, antifading with DAPI was used. Finally, we observed the slides under a fluorescence microscope (NIKON/Eclipse ci, Japan).^5^

#### 2.2.8 Electron microscopy of aorta

Targeted fresh tissues should be selected to minimize mechanical damage. The aortic root tissue was cut into 1 mm^3^, fixed and transferred into EP tubes with fresh TEM fixative (fixed at 4°C for preservation and transportation) for further fixation. Tissue samples were dehydrated at room temperature, and resin penetrated and embedded. Resin embedding models and samples were placed in an oven at 65°C for polymerization over 48 h. Remove the resin block from the embedding model and set aside at room temperature. The resin blocks were cut into 60–80 nm thin on the ultra‐microtome, and the tissues were fished out onto the 150 mesh cuprum grids with formvar film. Uranium acetate saturated alcohol solution (2%) avoided light staining for 8 min, rinsed in 70% ethanol for three times and then rinsed three times with ultra‐pure water. Stained with 2.6% lead citrate for 8 min, and then rinsed three times with ultra‐pure water. After dried by the filer paper, the cuprum grids were put no the grid board and dried overnight at room temperature. The cuprum grids were observed under TEM and took images.

#### 2.2.9 Cell culture

Cells‐mouse‐originated macrophages (RAW 264.7) were supplied by the iCell Bioscience Inc. Cells were cultured in Dulbecco's modified Eagle medium, supplemented with 10% fetal bovine serum and 1% penicillin/streptomycin, and incubated at 37°C in humidified 5% CO_2_. RAW 264.7 cells were used from passages 4–25. A medicated serum and a blank serum were prepared using 20 healthy SPF male Sprague–Dawley rats. The rats were randomly divided into two groups: the Huayu Qutan formula group and the control group. This division was done using the random number table method, with 10 rats in each group. In the medicated serum group, the rats were gavaged with Huayu Qutan Recipe at a dosage of 9.0 g/kg/d, while the rats in the blank serum group were given an equal volume of 0.9% saline. Serum was collected from the abdominal aorta after anaesthesia. After the serum was inactivated, sterilized with 0.22 um filter, stored at −20°C for spare and diluted to the required concentration with DMEM medium.

RAW 264.7 cells were divided into eight groups, and the experimental groupings were as follows: ①Control group (CTRL): RAW 264.7 cells were cultured normally without any treatment; ②Model group (MOD): RAW 264.7 cells were treated with 50 μg/mL ox‐LDL for 48 hours; ③Huayu Qutan Recipe group (HYQT): RAW 264.7 cells were treated with 50 μg/mL ox‐LDL and 10% rat serum (gavaged with the Huayu Qutan Recipe) for 48 hours; ④Huayu Qutan Recipe control group (HYQT‐C): RAW 264.7 cells were treated with 50 μg/mL ox‐LDL and 10% rat serum (without gavaged Huayu Qutan Recipe) for 48 hours; ⑤Inhibitor of lysosome group (CQ): RAW 264.7 cells were treated with 50 μg/mL ox‐LDL and 10% rat serum (gavaged with the Huayu Qutan Recipe) with chloroquine (50 μM) for 48 h^21^; ⑥mTOR agonist group (MHY1485): RAW 264.7 cells were treated with 50 μg/mL ox‐LDL and 10% rat serum (gavaged with the Huayu Qutan Recipe) with MHY1485 (100 nmol/L) for 48 hours^22^; ⑦Solvent of CQ, MHY1485, and rapamycin (DMSO): RAW 264.7 cells were treated with 50 μg/mL ox‐LDL and 0.1% DMSO for 48 h; ⑧Inhibitor of mTOR group (RAPA): RAW 264.7 cells were treated with 50 μg/mL ox‐LDL and rapamycin (50 nmol/L) for 48 h.^23^

#### 2.2.10 Q‐Orbitrap high‐resolution MS analysis

Serum (50 μL) was taken and then 200 μL methanol was added. Vortexed mixture 20,000 xg for 5 min, and centrifuged at 4°C for 10 min. Supernatant was analysed. The data collected by the high‐resolution liquid quality system were initially sorted through CD 2.1 (Thermo Fisher) and then searched and compared with the database (mzCloud and mzVault).

#### 2.2.11 Cell viability analysis

Cell counting Kit‐8 was used to detect the cell viability of RAW 264.7 cells. Cells were plated at a density of 2 × 10^5^ per well in 96‐well plates. After treatment with 4%, 6%, 8% and 10% HYQT medicated serum for 6 h, 12 h, 24 h and 48 h, 10 μL of CCK‐8 was added, and the 96‐well plates were incubated 2 h at 37°C in incubator. Untreated cells served as negative controls. OD was measured at 450 nm using microplate readers from BioTek/Epoch. Results were expressed as a percentage of untreated control cell measurements.

#### 2.2.12 ORO staining

RAW 264.7 cells were fixed in 4% Paraformaldehyde (PFA) and rinsed three times with phosphate‐buffered saline (PBS) (Servicebio, Lot.G0002); stained with ORO at room temperature for 30 min. The staining was evaluated by optical microscope and quantified by Image J software.^24^

#### 2.2.13 Analysis of cholestery ester (CE) content

Measurement of lipid levels using the free cholesterol (FC) content detection kit and the total cholesterol (TC) test kit. According to the manufacture protocol, the levels of TC and FC were quantified. The difference between TC and FC was counted as the content of CE.^25^

#### 2.2.14 BODIPY staining

LDs were stained by incubating the cells or sections with BODIPY 493/503 (MaoKangbio, Lot. 1912X200515) for 30 min. Then immunofluorescence was performed. The staining was assessed by fluorescence microscope. Image J software was used for quantitative analysis.

#### 2.2.15 LysoTracker red staining

After the cells received the specified treatment, lysosomes in living cells were labelled with LysoTracker Red at 75 nM and 37°C for 60 min according to the manufacturer's protocol. Image J software was used for quantitative analysis.

#### 2.2.16 Immunofluorescence

LC3b was co‐stained with lipid droplets. The 6‐well plates were then removed, absorbed the culture medium, added an appropriate amount of BODIPY dye to the final concentration of 1 μmol/L and kept them in a dark place for 10 min. The dye solution was removed and cleaned with PBS once, and cells were fixed with 4% paraformaldehyde for 15 min. Cell slides were washed with PBS three times, dried and sealed with 6% goat serum for 30 min. Anti‐LC3b antibody (1:200) was added and incubated overnight at 4°C. It was then incubated with DyLight 954‐labelled II antibody (1:500) at 37°C for 60 min. Washed PBS three times for 3 min each, then dropped Dapi‐Fluoromount‐G. The collected images were observed under a fluorescence microscope.

#### 2.2.17 Transmission electron microscopy of RAW 264.7 cells

Cells were precipitated by centrifugation to obtain cell pellet the ice‐cold 1% agarose solution was prepared by heating and dissolving in advance, then added to the EP tubes. Cell pellet were encapsulated in agarose. Agarose blocks were protected from light and fixed in 1% OsO_4_ for 2 h at room temperature. OsO_4_ was removed and cells were rinsed three times with 0.1 M PB (pH 7.4) for 15 min each. Samples were dehydrated at room temperature. Resin blocks were cut into 60–80 nm slices and placed on a 150‐mesh cup grid with formvar film to observe and capture images after staining.

#### 2.2.18 Cholesterol efflux assay

RAW264.7 cells were incubated with NBD‐cholesterol for 4 h. After cholesterol loading, the cells were washed and incubated with phenol red free Dulbecco's modified Eagle and HDL medium for 4 h. The wells only treated with medium were used as control. The fluorescence intensity of cell lysate and cell culture supernatant was determined by microplate reagent. The rate of efflux was calculated as follows: Efflux rate (%) = medium counts/ (medium counts + cell lysate counts) × 100%.^26^, ^27^

#### 2.2.19 Nuclear localization of TFEB

Cell slides were fixed with 2% paraformaldehyde for 10 min; 0.2% TritonX‐100 permeable membrane for 10 min; wash with 10 μmol·L^−1^PBS for 3–5 min; 5% BSA at room temperature for 1 h; wash with 10 μmol·L^−1^ PBS for 2 × 5min; Rabbit anti‐TFEB (1:50) was incubated at room temperature for 2 h. Washed with 10 μmol·L^−1^ PBS for 3–10 min; Goat anti‐rabbit IgG‐Alexa 594 (1:500) was protected from light and moisturized at room temperature for 1 h. Washed with 10 μmol·L^−1^ PBS for 4–5 min. The anti‐fluorescence quenching agent containing DAPI was sealed and placed in an inverted fluorescence microscope to collect images. Fluorescence quantitative analysis was performed by ImageJ software.

#### 2.2.20 Quantitative real‐time polymerase chain reaction (qRT‐PCR)

Total RNA was extracted from RAW264.7 cell lines using Trizol total RNA isolation reagent according to the manufacturer's instructions, it was extracted from the cell lines using Trizol total RNA separation reagent and treated with Turbo DNase. cDNA was synthesized from total RNA (0.5 mg) using cDNA reverse transcription kit. After the primers were designed (Table 3), real‐time PCR was performed. It was performed on 7500 Fast real‐time PCR System (Applied Biosystem). DD threshold cycle (Ct) method was used to calculate the gene expression in each sample, and the average of three independent analyses was used to normalize to the endogenous control gene GAPDH.

**TABLE 3:** A list of the primers used in the qRT‐PCR.

Beclin1	Forward	CCGTACAGGATGGACGTGGA
	Reverse	TGGGTTTTGATGGAATAGGAGC
P62	Forward	CACTACCGCGATGAGGATGG‐3
	Reverse	CTGCACTTATAGCGAGTTCCCAC
ABCA1	Forward	GATGGCAATCATGGTCAATGG
	Reverse	AGCTGGTATTGTAGCATGTYCCG
ABCG1	Forward	AGGTCTCCAATCTCGTGCCG
	Reverse	GCGACTGTTCTGATCCCCGT
SCARB1	Forward	CTTGCTGCTGAGGGAGTCTCG
	Reverse	CTGAAGGAGACGGAGACAGAGG
Lamp‐1	Forward	CAGCAGGCCTTGCACAT
	Reverse	GTAGGTGGTCAGAAAGGAGG
GAPDH	Forward	CCTCGTCCCGTAGACAAAATG
	Reverse	TGAGGTCAATGAAGGGGTCGT

#### 2.2.21 Western blot analysis

RIPA Lysis Buffer was used to obtain total protein from RAW 264.7 cells. The Enhanced BCA Protein Assay Kit was adopted to determine protein concentrations. SDS‐polyacrylamide gel was used to separate cell proteins by electrophoresis and transferred to PVDF membranes. PVDF membranes were incubated overnight at 4°C with the following primary antibodies: Beclin1 (1:1000), LC3I/II (1:1000), p62 (1:1000), ABCA1 (1:1000), p‐mTOR (1:1000) and mTOR (1:1000), ABCG1 (1:1000), SCARB1 (1:1000); membranes were washed with TBST and incubated with the secondary antibody β‐Actin (1:1000) for 2 h. According to the manufacturer's instructions, the ECL Ultra method was used to detect a DNR bioimaging system. All the experimental data were repeated three times independently.

#### 2.2.22 Molecular docking

The crystal structure of mTOR protein and the 3D structure of seven core compounds were obtained from PDB database (www.rcsb.org) and PubChem database (pubchem. ncbi. nlm. nih. gov). Then, obtained the low energy conformation of the core compounds by Chem3D 19.0 software. The water molecule and small molecule ligand were removed from the protein structure using Pymol software, and the protein structure and small molecule of the core component were respectively imported into AutoDock1.5. 6 software for analysis and processing, and Pymol software was used for visual analysis. The binding energy is calculated. The binding energy <0 kca/mol indicates that the docking can be done in the natural state, and the binding energy <−1.2 kca/mol indicates that the docking result is stable.

### 2.3 Statistical analysis

Results were shown as mean ± SD, and analysed by one‐way analysis of variance (ANOVA) with Tukey's post hoc test performed using GraphPad Prism Software 5.0. The cell counting and fluorescence intensity were analysed using Image J software. Differences of *P* < 0.05 was considered significant, statistical tests for each experiment are depicted in the relevant figure legends.

## 3 RESULTS

### 3.1 HYQT anti‐atherosclerosis

**FIGURE 1:**
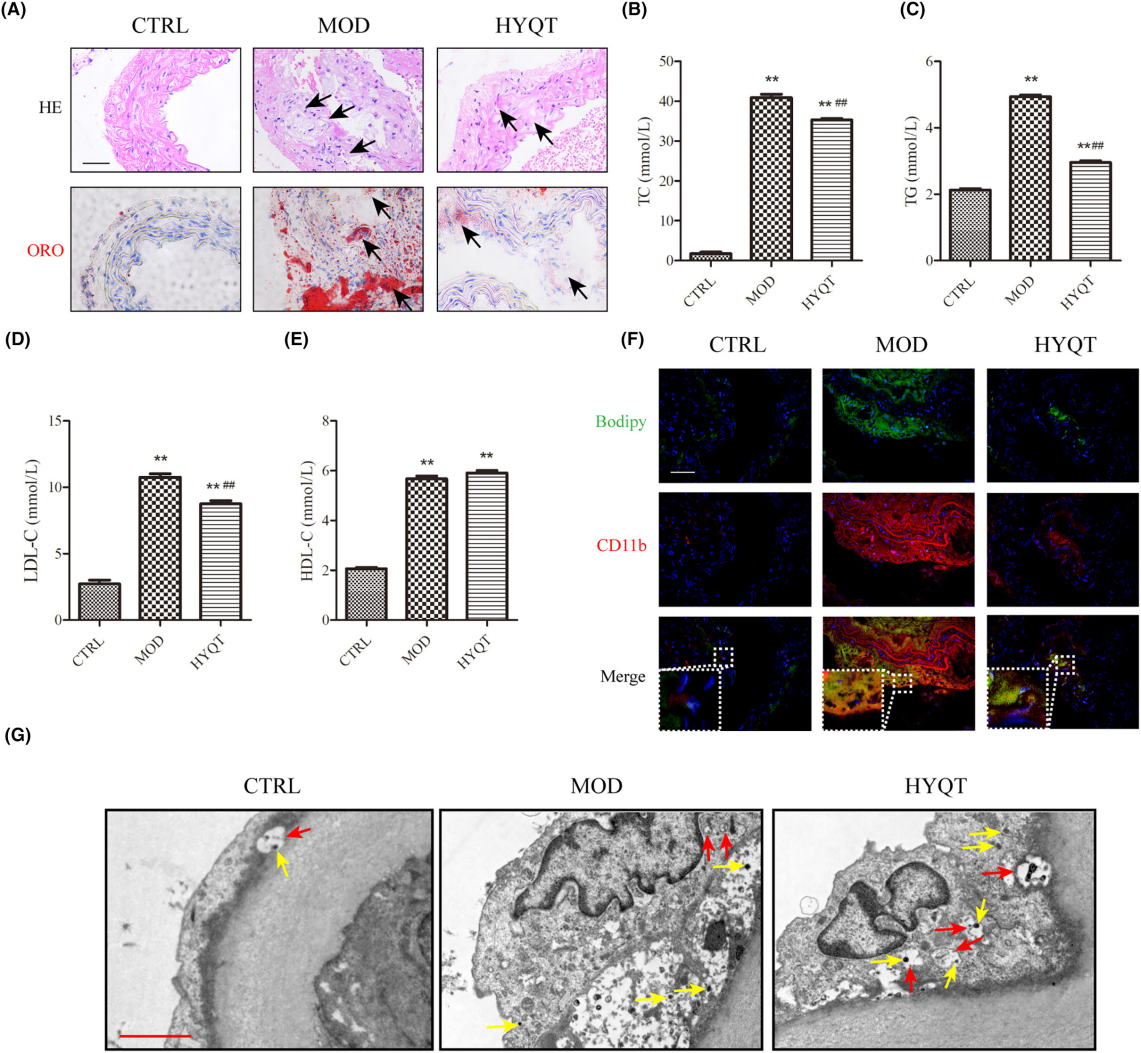
HYQT anti‐atherosclerosis. (A) HE and ORO staining in aorta (Scale bar: 100 μm, black arrow indicates lesion part); (B–E) The level of blood lipid (TC, TG, LDL‐C and HDL‐C); (F) The co‐localization of CD11b and BODIPY; (G) Transmission electron microscopy showed lysosome and autophagosome in the aorta. *P < 0.05, **P < 0.01 versus CTRL, ^#^ *P* < 0.05, ^##^ *P* < 0.01 versus MOD. (Scale bar: 2 μm, yellow arrow indicates lysosome, red arrow indicates autophagosome).

The pathological changes in the aortic root were observed by HE staining. There was no obvious formation of atherosclerotic plaque in the CTRL group. There was no obvious foam cells or lipid deposition. The intima was intact, and the elastic fibres were continuous. The aorta intima in MOD group displayed serious lesions. Lumen stenosis, intimal thickening, surface vitreous degeneration, atherosclerotic plaque and plaque deposition, destruction of internal elastic plates and breakage were observed. Lipids, foam cells, proliferating fibrous tissue or cholesterol crystals could be observed. HYQT alleviates pathological changes in the aorta. The ORO result showed a large area of ORO staining in the MOD group. The HYQT group had less ORO staining area (Figure 1A).

### 3.2 HYQT regulates blood lipid levels

Compared with the CTRL group, the TC, TG, LDL‐C and HDL‐C in the MOD group increased significantly. Compared with the MOD group, the levels of TC, TG and LDL‐C in the HYQT group were significantly decreased, and the level of HDL‐C did not change significantly (Figure 1B–E).

### 3.3 High‐fat diet induces accumulation of macrophage lipids in atherosclerosis and the effect of HYQT

CD11b and BODIPY were marked to observe the ratio of co‐staining macrophages to lipids in plaque (Figure 1F). Similar to the ORO staining results, immunofluorescence showed that the positive area of the aortic roots in the CTRL group was hard to observe. Macrophage adhesion and lipid droplet accumulation was rare in the aorta. CD11b and BODIPY staining with different fluorescence intensities were observed in the MOD, HYQT group. The MOD group has a higher level of co‐localization. However, the HYQT group reduced.

### 3.4 HYQT promotes the formation of autophagosomes

The autophagosomes in the aortic plaque were detected by transmission electron microscopy (Figure 1G). We observed no macrophage accumulation around the endothelium in the CTRL group. Macrophage attachment in the MOD group, fewer lysosomal vesicles and fewer autophagosomes. A large number of autophagosomes were observed in the HYQT group, and more lysosomes were found.

### 3.5 HYQT inhibits formation of foam cell in RAW 264.7 cells

**FIGURE 2:**
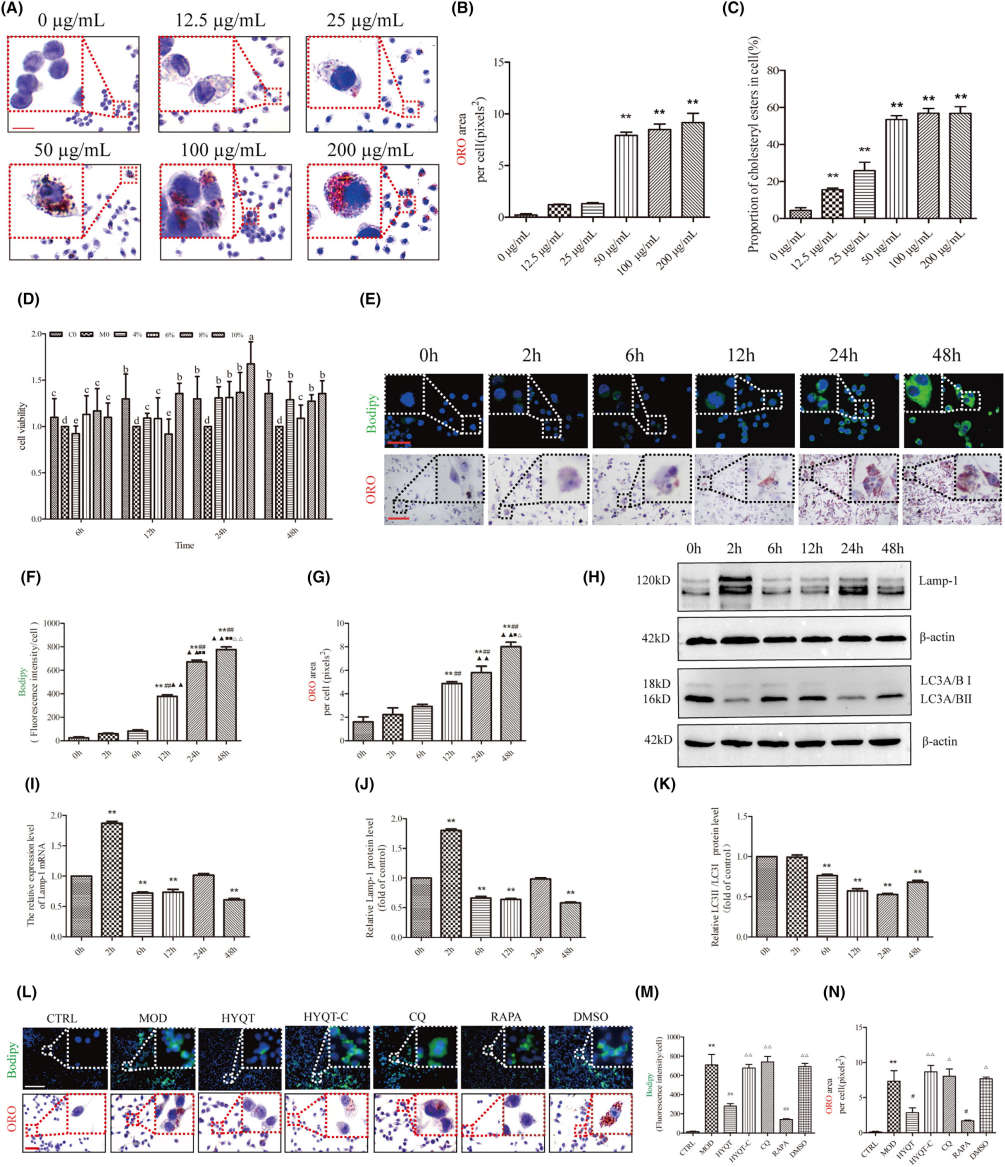
The efficacy of HYQT on LDs accumulated. (A) For ORO, RAW 264.7 cells were treated with different concentrations of oxLDL (Scale bar: 100 μm); (B) ORO image analysis (B *n* = 18 per group); (C) and CE level (C *n* = 6 per group); (D) Cells activity with different concentrations of HYQT serum and different incubation time. The column diagrams marked by different letters were significant to each other. Values were mean ± SD (D *n* = 3 per group); RAW 264.7 cells were treated with ox‐LDL and HYQT, then treated with CQ for 0, 2, 6, 12, 24 and 48 h. (E) The BODIPY and ORO staining were used. (F,G) Quantitative statistics of BODIPY and ORO staining. (H) Lamp‐1 and LC3II/I protein were detected. (I) Lamp‐1 mRNA, (J) Lamp‐1 protein, (K) LC3II/I protein. (L) BODIPY and ORO stain (Scale bar: 200 μm, 100 μm); (M) BODIPY image analysis (G *n* = 13 per group); (N) ORO image analysis (F *n* = 16 per group); **P < 0.01 versus CTRL, ^#^ *P* < 0.05, ^##^ *P* < 0.01 versus MOD, ^△^ *P* < 0.05, ^△△^ *P* < 0.01 versus HYQT. Bars represent the mean of the group ± SD (*n* = 5). Columns with different superscripts (a, b, c, d, and e) are significantly different (*P* < 0.05). CTRL: control, MOD: model, HYQT: HYQT medicated serum, HYQT‐C: control serum. CQ: treated with CQ (50 μM for 48 h), RAPA: treated with RAPA (50 nmol/L for 48 h), DMSO: treated with DMSO (0.1% DMSO for 48 h). ORO: oil Red O stain; BODIPY: fluorescent probe of lipid droplets.

RAW 264.7 cells were induced by ox‐LDL, macrophage‐derived foam cell formation and cholesterol efflux were detected, and the efficacy of HYQT was determined. RAW 264.7 treated with different concentrations of ox‐LDL for 48 h. Stimulated macrophages with ox‐LDL at different concentrations (0, 12.5, 25, 50, 100 and 200 μg/mL for ORO staining). The ORO staining area was analysed, which did not significantly chang between 0 and 12.5, 25 μg/mL. There was less ORO staining area treated with 12.5, 25 μg/mL ox‐LDL. The area was not significantly increased compared with the 0 ug/mL ox‐LDL group. After treatment with 50 μg/mL ox‐LDL, an obvious ORO staining area was observed, and cell morphology was significantly changed compared with 0 μg/mL. Compared with the 0 ug/mL ox‐LDL group, the ORO staining area was significantly increased. A large area of ORO staining was observed at 100 μg/mL and 200 μg/mL. A significant change in cell morphology was observed, more pseudopodia appeared. Compared with the 0 ug/mL ox‐LDL group, the area of ORO staining was significantly increased (Figure 2A,B).

Then, CE was detected. The results showed that with the increase of ox‐LDL concentration, intracellular lipid droplets increased. Treated with 12.5, 25, 50, 100 and 200 ug/mL ox‐LDL, CE content were significantly increased. When treated with 50 ug/mL ox‐LDL, more than 50 percent of CE in cells. 50 ug/mL ox‐LDL was selected for the subsequent experiments based on the result of ORO staining (Figure 2C).

Foam cells were inducted by ox‐LDL, then treated with different concentrations of HYQT medicated serum (4%, 6%, 8% and 10%) and at different times (6 h, 12 h, 24 h and 48 h). The CCK‐8 results showed cells are most active at 24 h when the concentration of HYQT‐medicated serum is 10%. Therefore, cells were treated with 10% medicated serum for 24 h for subsequent experiments (Figure 2D).

Chloroquine (CQ) was used to identified the role of autophagy in lipid droplet accumulation. CQ intervention in different time periods had an effect on the level of autophagy. RAW 264.7 was treated with CQ 0 h, 2 h, 6 h, 12 h, 24 h and 48 h. Then, BODIPY staining and ORO staining was detected to assess the lipid droplet accumulation (Figure 2E). LC3A/BII, LC3A/BI, Lamp‐1 (the key lipophagy protein of lysosomal pathway), protein expression level were detected to assess the autophagy flow. The results showed that there was a significant increase in intracellular lipid droplet accumulation at 12 h. A significant increase in BODIPY staining at 12 h compared to 0 h, 2 h and 6 h, and a significant increase in ORO staining at 12 h compared to 0 and 2 h (Figure 2F,G).

The Lamp‐1 mRNA and protein levels were significantly increased at 2 h compared to 0 h, and subsequently showed significant decreases at 6, 12, 24 and 48 h compared to the 0 h. Similarly, LC3II/I protein level was significantly decreased at the time points (6, 12, 24 and 48 h) compared to the 0 h. Based on the results of BODIPY and ORO staining as well as lamp‐1 and LC3II/Iprotein expression analysis, RAW264.7 cells treated with CQ for a duration of 48 h were selected for subsequent experiments (Figure 2H–K).

RAW 264.7 cells stained with ORO and BODIPY, when treated with different factors. The results showed that the accumulation of lipid droplets was different in the CTRL, MOD, HYQT, HYQT‐C, CQ, DMSO and RAPA groups. In the CTRL group, no lipid droplets were detected, and the cells appeared round or oval. The MOD, HYQT, HYQT‐C, CQ, DMSO and RAPA groups exhibited varying levels of red staining and BODIPY fluorescence (Figure 2L). Compared with the CTRL group, a large amount of lipid deposition was observed in the MOD group, and the BODIPY fluorescence intensity and area of ORO in single cells were significantly increased. Compared with the MOD group, BODIPY fluorescence intensity and area of ORO in single cells in the HYQT and RAPA groups were decreased. Compared with the HYQT group, BODIPY fluorescence intensity and area of ORO in single cells in the HYQT‐C group showed a significant increase. Similarly, the CQ group also exhibited an increase in BODIPY fluorescence intensity and area of ORO compared with the HYQT group. Additionally, the DMSO group showed a significant increase in both BODIPY fluorescence intensity and area of ORO in single cells when compared with the HYQT group (Figure 2M,N).

### 3.6 HYQT promotes lipid degradation through the autophagy–lysosome pathway in foam cells

**FIGURE 3:**
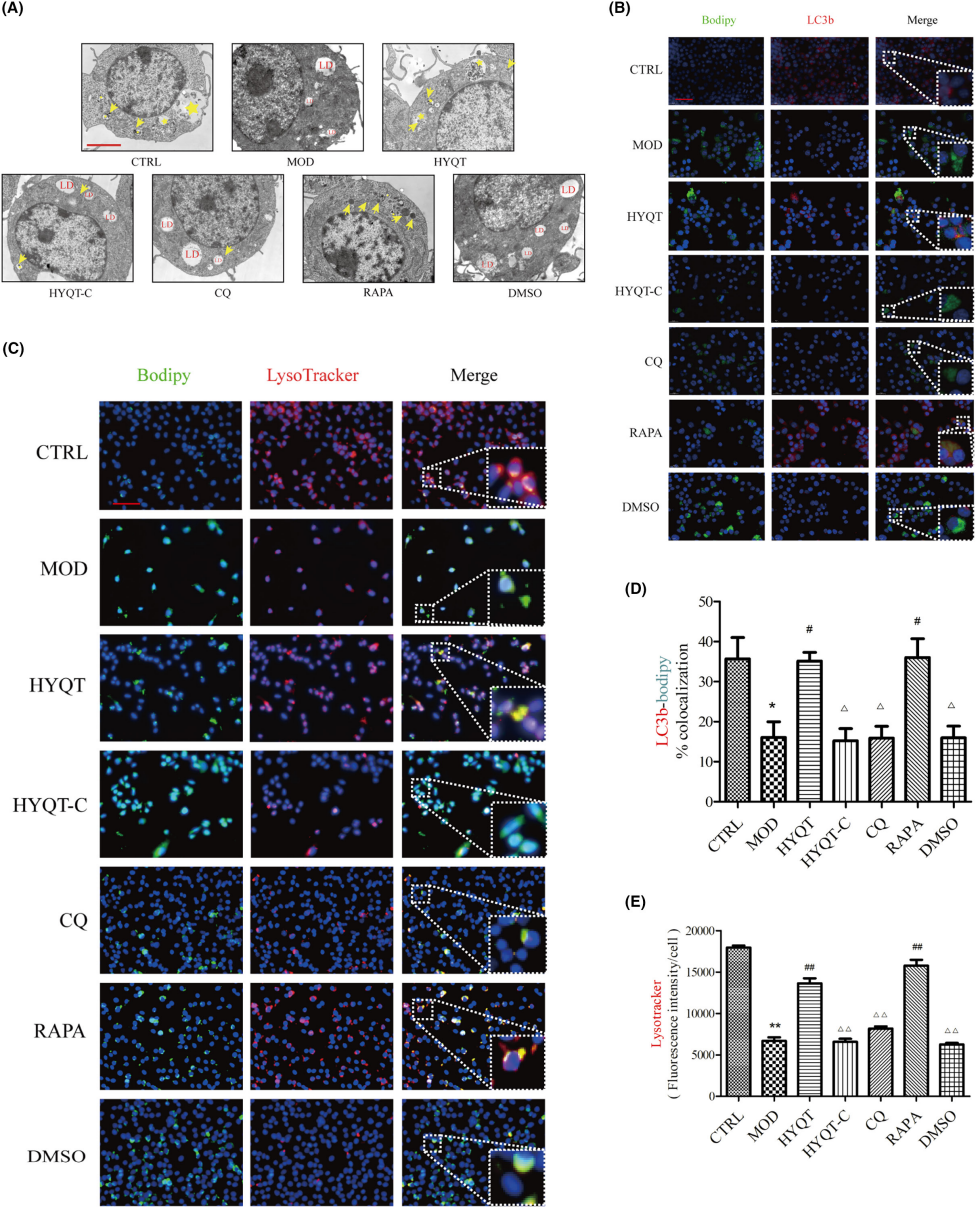
HYQT promoted lipophagy, CQ decreased the efficacy of HYQT on lipophagy. (A) Transmission electron microscope detected RAW 264.7 cells (arrow points (yellow) to the lysosome and the stars (yellow) locat lipophagy); LD (red): lipid drops, (Scale bar: 1 μm); (B) Fluorescence images of BODIPY‐LC3b (B Scale bar: 50 μm); (C) Fluorescence images of BODIPY‐LysoTracker (Scale bar: 100 μm); (D) Ratio co‐co‐localization of BODIPY‐LC3b (D *n* = 19 per group); (E) LysoTracker fluorescence intensity (E *n* = 16 per group). *P < 0.05, **P < 0.01 versus CTRL, ^#^ *P* < 0.05, ^##^ *P* < 0.01 versus MOD, ^△^ *P* < 0.05, ^△△^ *P* < 0.01 versus HYQT. LDs were stained with BODIPY (green), nuclei were stained with DAPI (blue), and lysosomes were stained with LysoTracker (red).

To further characterize the status of lipophagy flux, transmission electron microscopy (TEM) was used to determine the autophagosomes (Figure 3A). LDs were readily identifiable as round light‐density structures that were not limited by a bilayer lipid membrane.^28^, ^29^ The results showed that the number and area of LDs per cell increased in ox‐LDL‐treated group while decreasing in the HYQT group. Additionally, autolysosome and lysosome numbers increased in the HYQT and RAPA groups. However, these were reversed by CQ.

The ratio of LC3b‐BODIPY co‐localization and labelled lysosomes with LysoTracker was analysed in order to explain the occurrence of lipophagy in macrophages (Figure 3B,C). In comparison to the CTRL group, the MOD group showed a significant decrease in the ratio of co‐localization, while the HYQT group demonstrated a significant increase in the ratio of co‐localization compared to the MOD group. Moreover, CQ reversed the effect of HYQT. Compared with the CTRL group, the co‐localization ratio of LC3b‐BODIPY in the MOD group was significantly decreased. Compared with the MOD group, the co‐localization ratio significantly increased when treated with HYQT. When compared with the HYQT group, the co‐localization ratio of LC3b‐BODIPY in the HYQT‐C, CQ and DMSO groups was significantly decreased (Figure 3D).

The fluorescence intensity of the LysoTracker showed the similar results were obtained. Compared with the CTRL group, the fluorescence intensity of the LysoTracker significantly decreased in MOD group. Compared with the MOD group, the fluorescence intensity of the LysoTracker significantly increased in HYQT and RAPA groups. Compared with the HYQT group, the fluorescence intensity of the LysoTracker significantly decreased in HYQT‐C, CQ and DMSO groups (Figure 3E).

### 3.7 HYQT regulates lipophagy genes and proteins

**FIGURE 4:**
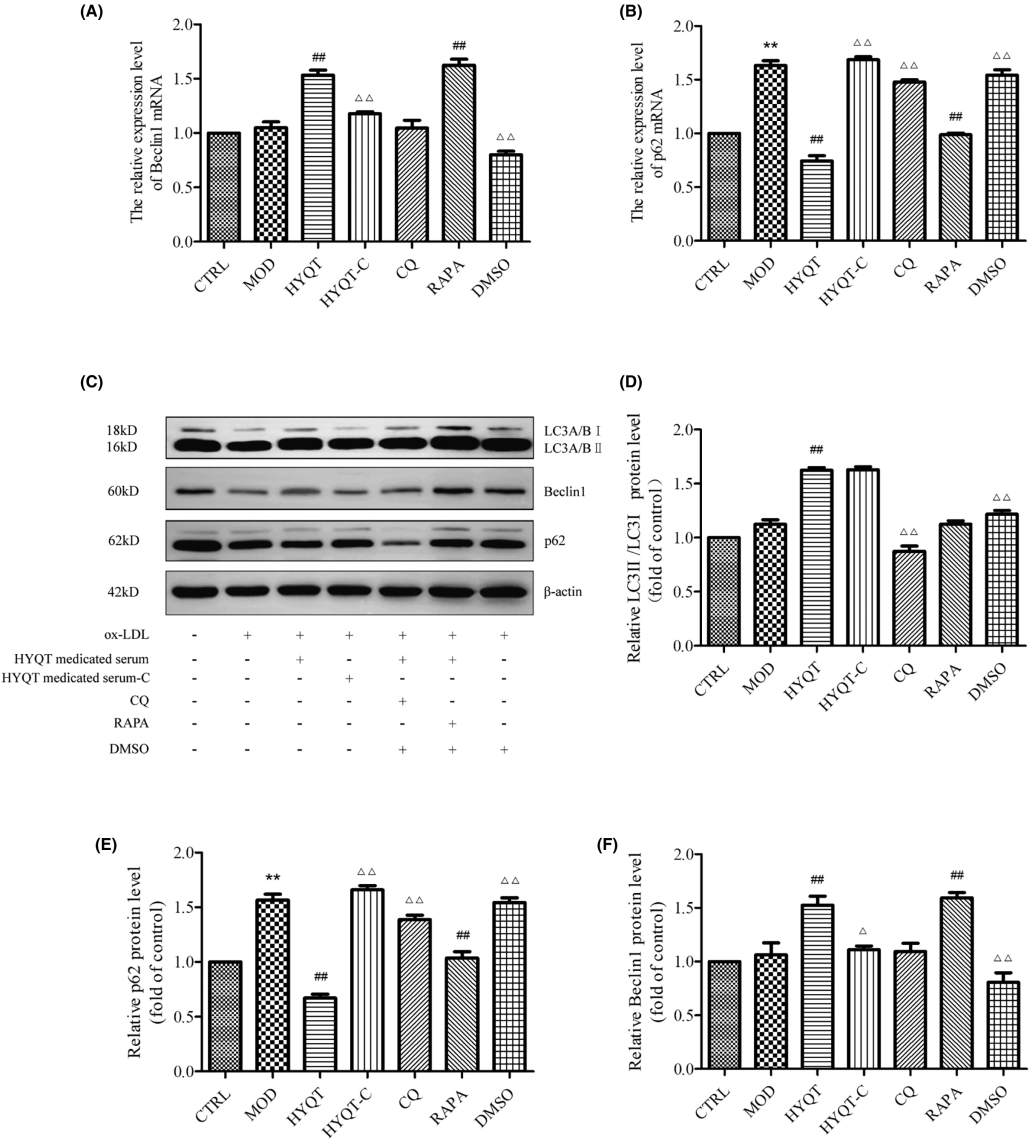
The expression of LC3II/I, Beclin1, p62 and RAPA and CQ impacted the expression of autophagy genes and proteins detected by qRT‐PCR and WB. (A) qRT‐PCR for Beclin1, and (B) p62 normalized ratios; (C) western blot for LC3, Beclin1 and p62 treated with different factors and normalized ratios (D–F). All data were presented as the mean ± SD (*n* = 3), *P < 0.05, **P < 0.01 versus CTRL, ^#^ *P* < 0.05, ^##^ *P* < 0.01 versus MOD, ^△^ *P* < 0.05, ^△△^ *P* < 0.01 versus HYQT.

The gene expression of cells was detected by qRT‐PCR. The expression of macrophage lipophagy‐related genes and proteins was detected. Compared with the CTRL group, Beclin1 mRNA in the MOD group was increased, but not significantly, and p62 mRNA expression was significantly increased. Compared with the MOD group, the relative expression of Beclin1 mRNA was significantly increased in HYQT, RAPA groups, and p62 mRNA was significantly decreased in HYQT, RAPA groups. Compared with HYQT group, the relative expression of Beclin1 mRNA in the HYQT‐C, CQ and DMSO groups was decreased, p62 mRNA in the HYQT‐C, CQ and DMSO groups was significantly increased (Figure 4A,B).

Compared with CTRL group, the protein expression of LC3II/I, Beclin1 in MOD group were not significantly, p62 protein decreased significantly in MOD group. Compared with the MOD group, the relative expression of LC3II/I proteins in the HYQT group was significantly increased, and Beclin1 protein increased in HYQT and RAPA group. While the relative expression of p62 protein was significantly decreased in HYQT and RAPA groups compared with the MOD group. Compared with the HYQT group, the relative expression of LC3II/I protein in the CQ group was decreased; the relative expression level of Beclin1 protein was significantly decreased in HYQT‐C and DMSO groups; and the expression of p62 protein was increased in HYQT‐C and DMSO groups (Figure 4C–F).

### 3.8 HYQT regulates mTORC1/TFEB signalling pathway and cholesterol efflux proteins

**FIGURE 5:**
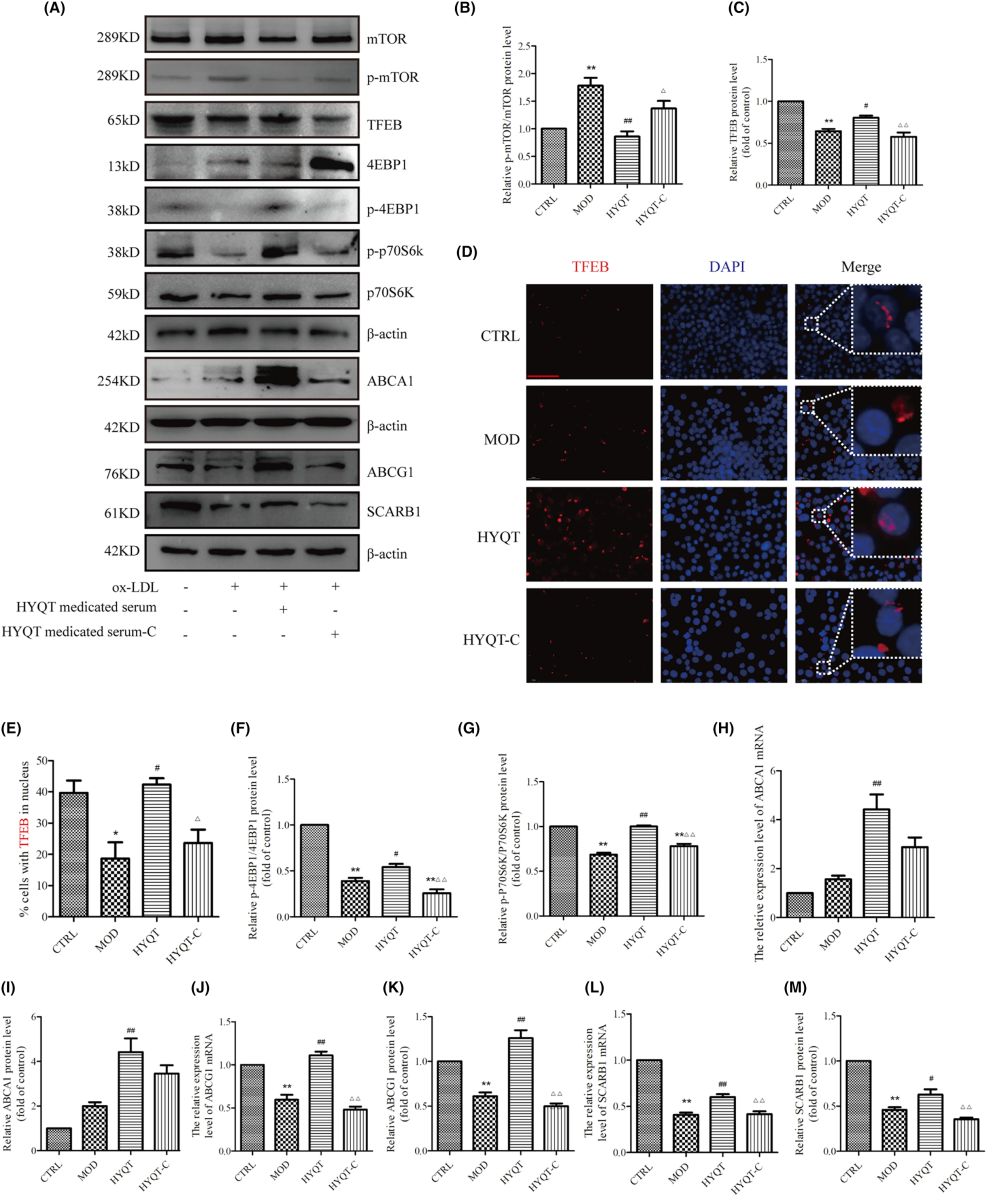
HYQT regulates the mTORC1/TFEB signalling pathway and cholesterol efflux proteins. (A) Detected the proteins expression by WB; (B) p‐mTOR/mTOR; (C) TFEB; (D) Immunofluorescence for TFEB (Scale bar: 50 μm); (E) Ratio of TFEB in nucleus (*n* = 12 per group). Normalized ratios of (F) p‐4EBP1/4EBP1, (G) p‐P70S6K/P70S6K, (H) ABCA1 mRNA, (I) ABCA1 protein, (J) ABCG1 mRNA, (K) ABCG1 protein, (L) SCARB1 mRNA, (M) SCARB1 protein. qRT‐PCR and WB data were presented as the mean ± SD (*n* = 3). *P < 0.05, **P < 0.01 versus CTRL, ^#^ *P* < 0.05, ^##^ *P* < 0.01 versus MOD, ^△^ *P* < 0.05, ^△△^ *P* < 0.01 versus HYQT. TFEB proteins were stained with fluorescence (red), and nuclei were stained with DAPI (blue).

WB analysis was performed to detect the expression of the mTORC1/TFEB signalling pathway, mTOR/4EBP1/P70S6K signal pathway, ABCA1, ABCG1 and SCARB1 proteins level (Figure 5A). First, the level of p‐mTOR/ mTOR was analysed (Figure 5B). Compared with the CTRL group, the MOD group significantly increased, while compared with the MOD group, the HYQT group significantly decreased. Compared with the HYQT group, the HYQT‐C group significantly increased. The TFEB protein level decreased in MOD group compared with CTRL group. Compared with MOD group, HYQT group was increased, while HYQT‐C group decreased significantly, compared with HYQT group (Figure 5C). Moreover, the translocation to the nucleus is a prerequisite for TFEB to function, and TFEB activates downstream gene expression (Figure 5D). The data showed that, compared with the CTRL group, the nuclear TFEB ratio in the MOD group decreased, and increased in the HYQT group when compared with the MOD group. Compared with the HYQT group, the ratio in the HYQT‐C was significantly decreased (Figure 5E).

The mTOR/4EBP1/P70S6K signalling pathway protein were as follows. Compared with CTRL group, the p‐4EBP1/4EBP1 protein level was decreased in MOD group. Compared with MOD group, p‐4EBP1/4EBP1 protein level was increased in HYQT group. Compared with HYQT group, HYQT‐C group was decreased significantly. The p‐P70S6K/P70S6K protein level was decreased in MOD group, compared with CTRL group. Compared with the MOD group, p‐P70S6K/P70S6K protein level was increased in HYQT group. Compared with HYQT group, p‐P70S6K/P70S6K protein level in HYQT‐C group was decreased significantly (Figure 5F,G).

The expression of the ABCA1 mRNA and protein was as follows (Figure 5H,I). Compared with the CTRL group, the MOD group was not changed significantly; compared with the MOD group, the HYQT group significantly increased; compared with the HYQT group, the HYQT‐C group significantly decreased. The expression of the ABCG1 mRNA and protein was as follows (Figure 5J,K). Compared with the CTRL group, the MOD group significantly decreased; compared with the MOD group, the HYQT group significantly increased; compared with the HYQT group, the HYQT‐C group significantly decreased. The expression of the SCARB1 mRNA and protein was as follows (Figure 5L,M). Compared with the CTRL group, the MOD group significantly decreased; compared with the MOD group, the HYQT group significantly increased; compared with the HYQT group, the HYQT‐C group significantly decreased.

### 3.9 HYQT regulates cholesterol efflux via mTOR/TFEB signalling pathway

**FIGURE 6:**
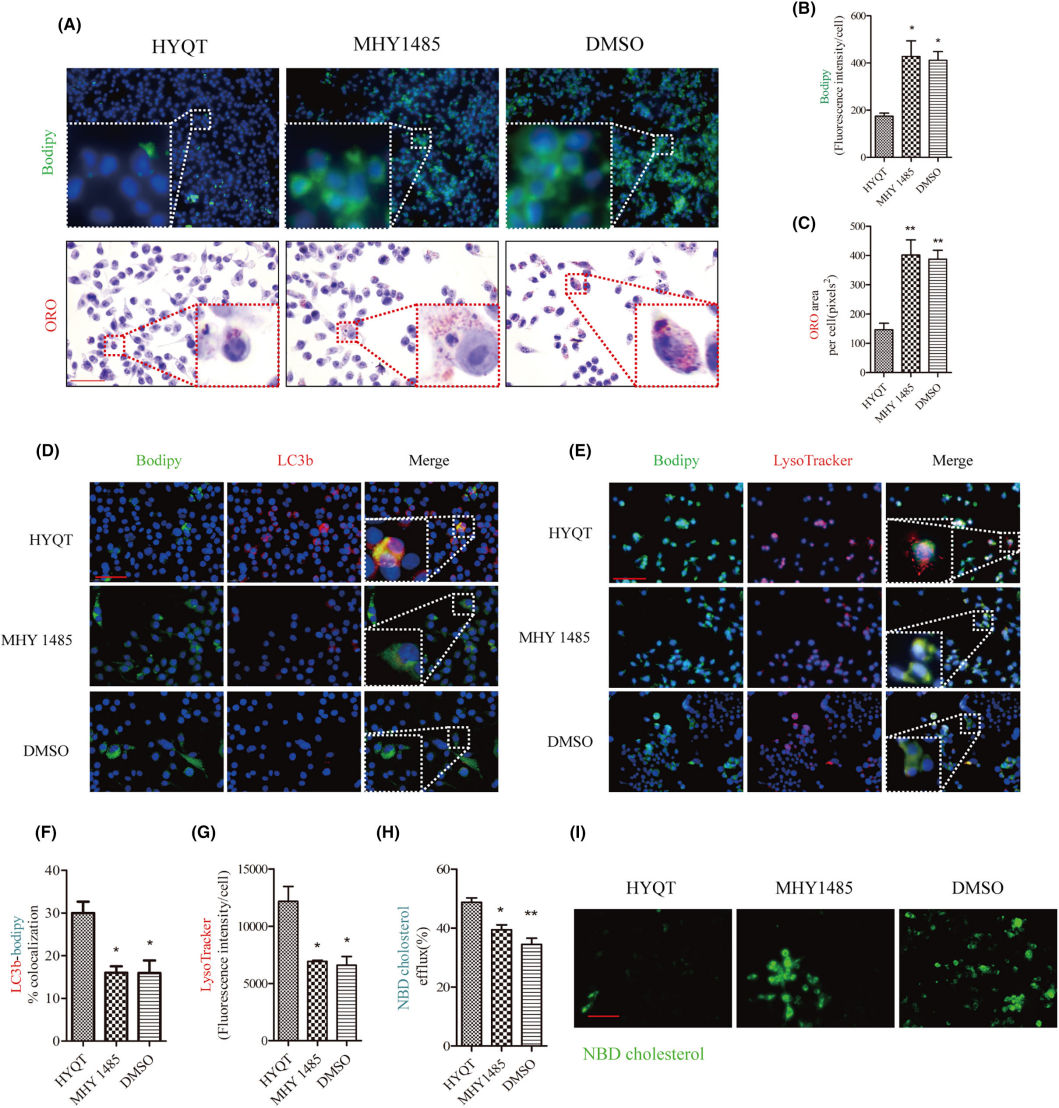
mTORC1/TFEB signalling pathway had an influence on lipophagy and cholesterol efflux. (A) Cholesterol levels of per cell treated with MHY1485 (A *n* = 15 per group, BODIPY Scale bar: 200 μm, ORO Scale bar:100 μm) and (B) analysed fluorescence intensity of BODIPY and (C) ORO pixels^2^; (D) Fluorescence images of BODIPY‐LC3b (Scale bar: 50 μm); (E) Fluorescence images of BODIPY and lysosome probe (Scale bar: 100 μm); (F) ratio of co‐localization of two (F *n* = 19 per group); (G) LysoTracker fluorescence intensity (G *n* = 16 per group); (H) Ratio of NBD cholesterol efflux (H *n* = 6 per group). (I) NBD cholesterol image treated with MHY1485 (Scale bar: 500 μm). *P < 0.05, **P < 0.01 versus HYQT. MHY1485 (100 nmol/L for 48 h). ORO: oil red O stain; BODIPY: fluorescent probe of lipid droplets intracellular cholesterol was stained with NBD 493/503 (green).

To clarify the effect of mTOR/TFEB on cholesterol efflux, MHY1485 (the agonist of mTOR) was applied. First, to assess the effect of MHY1485 on lipid accumulation, BODIPY and ORO staining were employed (Figure 6A). The results suggested that MHY1485 promoted the lipid accumulation of RAW264.7 cells. Compared with the HYQT group, the BODIPY fluorescence intensity and ORO area of per cell in MHY1485 and DMSO groups increased significantly, and the same result was given in DMSO group (Figure 6B,C). To explore whether mTOR is involved in lipophagy, lysosome, LC3b and LDs were marked by fluorescence probe. The results found that the co‐localization of BODIPY and LC3b in MHY1485 and DMSO groups decreased significantly compared with the HYQT group (Figure 6D,F), and the per cell fluorescence intensity of the lysosome decreased in MHY1485 and DMSO groups compared with HYQT group (Figure 6E,G). Meanwhile, cholesterol efflux level of RAW264.7 cells was observed when treated with MHY1485. The results showed that, compared with the HYQT group, the fluorescence intensity of NBD cholesterol in the MHY1485 and DMSO groups were significantly increased (Figure 6I). The ratio of cholesterol efflux detected by the fluorescein micrometre was significantly decreased in the MHY1485 and DMSO groups compared to the HYQT group (Figure 6H).

**FIGURE 7:**
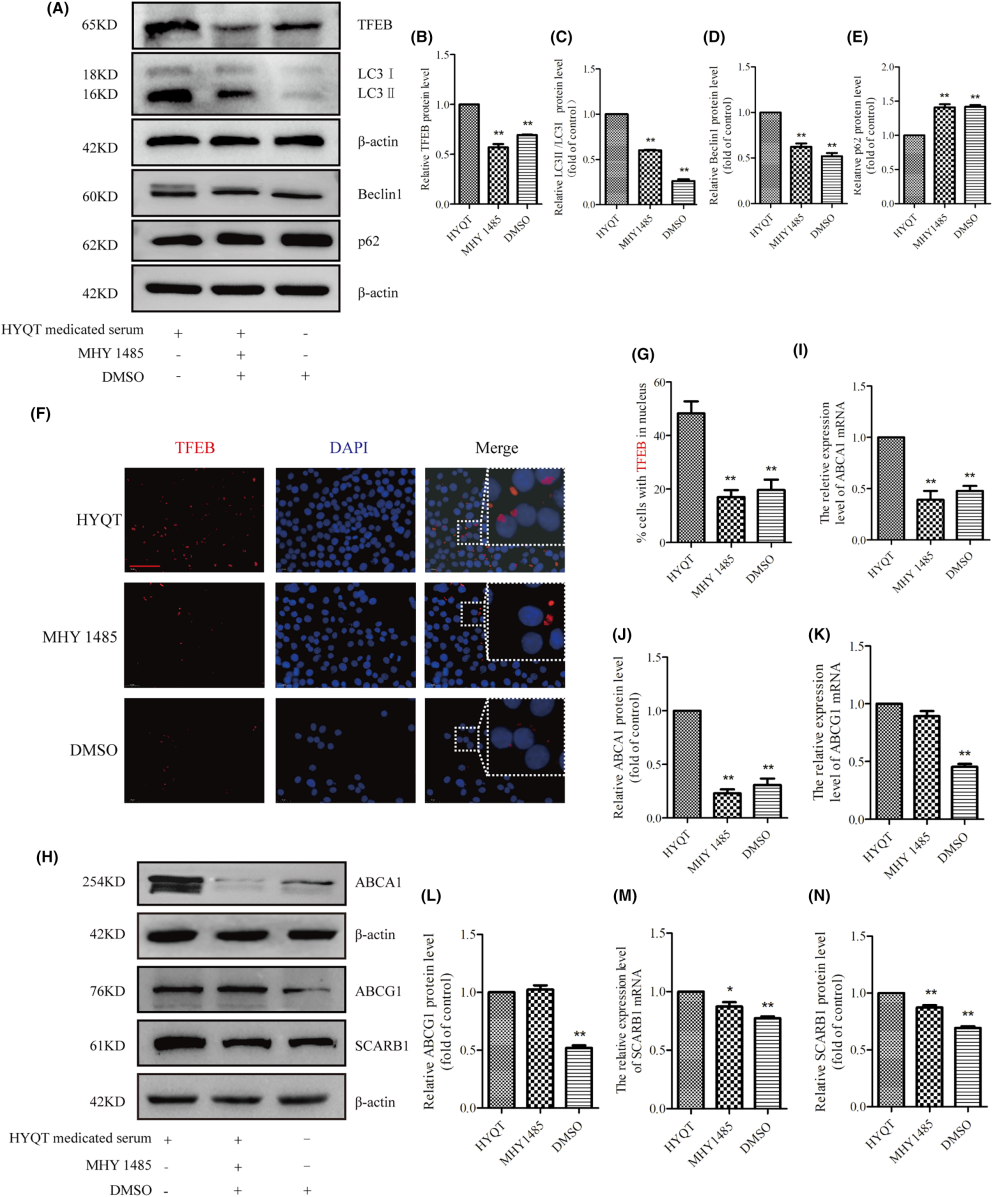
HYQT regulates lipophagy and cholesterol efflux via the mTORC1/TFEB/ABCA1‐SCARB1 signal axis. (A) TFEB, LC3II/I, Beclin1, and p62 proteins expression treated with MHY1485 and DMSO, and (B–E) normalized ratios; (F) Immunofluorescence for TFEB (Scale bar: 50 μm); (G) Ratio of TFEB in nucleus (*n* = 12 per group); (H) ABCA1, ABCG1 and SCARB1 proteins expression treated with MHY1485 and DMSO; (I–J) ABCA1, (K,L) ABCG1, (M,N) SCARB1 mRNAs and proteins normalized ratios. qRT‐PCR and WB data were presented as the mean ± SD (*n* = 3). *P < 0.05, **P < 0.01 versus HYQT. TFEB proteins were stained with fluorescence (red), and nuclei were stained with DAPI (blue).

WB analysis of TFEB, LC3I/II, Beclin1 and p62 protein expression (Figure 7A). The results showed that MHY1485 inhibited the TFEB, LC3II/LC3I and Beclin1 proteins (Figure 7B–D), and increased p62 protein expression (Figure 7E). DMSO has not promoted the autophagic flow in this study. The immunofluorescence was performed to detect the nuclear translocation of TFEB (Figure 7F). Results showed that the ratio of TFEB in the nucleus decreased in MHY 1485 and DMSO groups (Figure 7G).

Finally, ABCA1, ABCG1 and SCARB1 mRNA and protein expression were detected via qRT‐PCR and WB (Figure 7H). The results showed that ABCA1 mRNA and protein decreased after treated with MHY1485, and ABCA1 in DMSO group decreased compared with HYQT group (Figure 7I,J), but the ABCG1 mRNA and protein had no change after treated with MHY1485. ABCG1 expression level in DMSO group decreased significantly (Figure 7M,N). SCARB1 mRNA and protein decreased in MHY1485 group. DMSO (solvent of MHY 1485) has no effect on the results. The mTORC1/TFEB signalling pathway may be a regulator of ABCA1 and SCARB1, but not ABCG1. HYQT may regulate ABCG1 in other signalling pathways.

### 3.10 Q‐Orbitrap high‐resolution MS analysis the serum of HYQT

**FIGURE 8:**
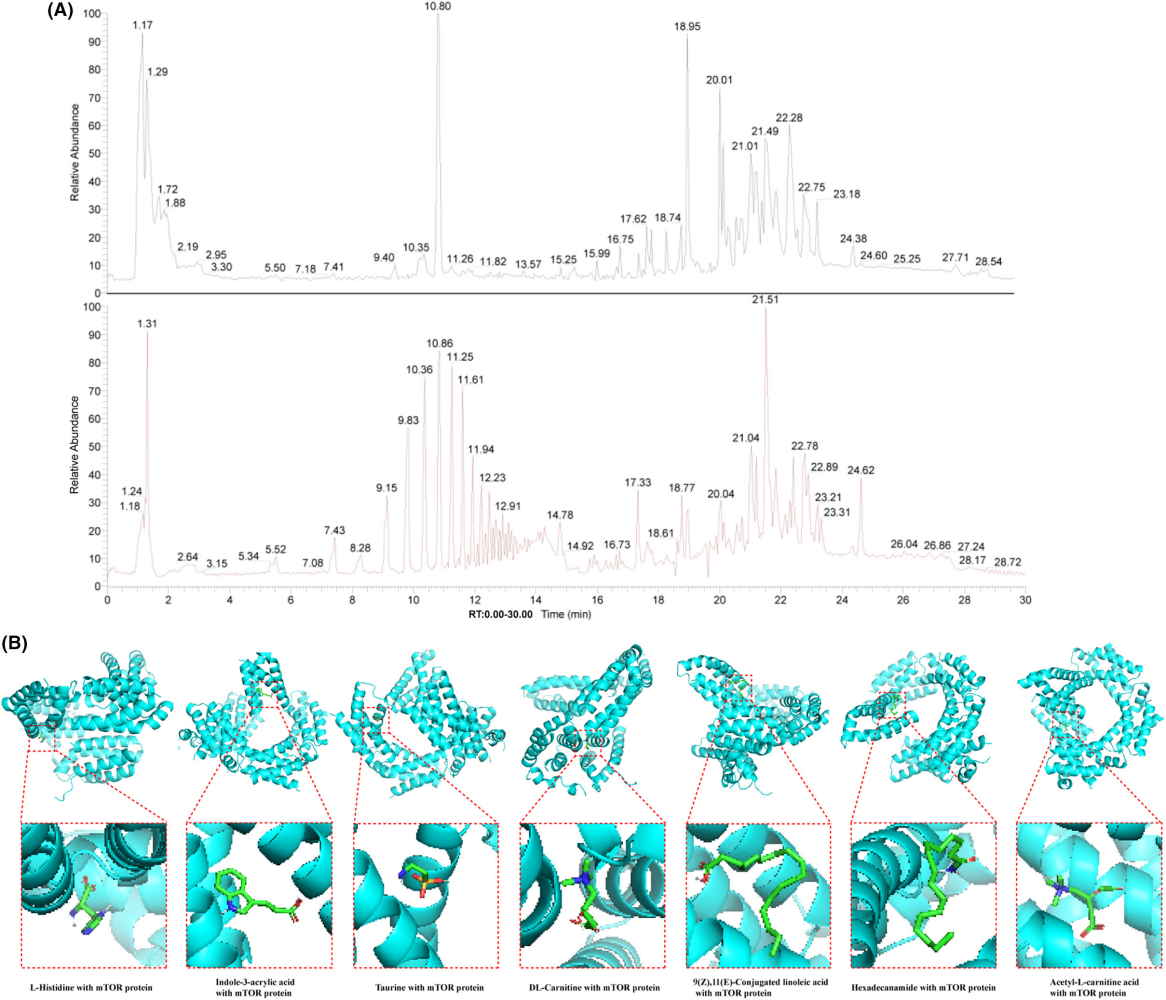
Total‐ion chromatogram by Q‐Orbitrap high‐resolution MS analysis of HYQT medicated serum and seven compounds in HYQT medicated serum bind with mTOR protein. (A) Detected in positive ion mode (red), detected in negative ion mode (black). (B) Structure diagram of compound binding to protein.

The medicated serum were matched with a total of 576 compounds in mzCloud. Analysed the structure of the analyte identified by the mass spectrum and identified 11 main compounds with a score ≥90 by mzCloud and mzVault, and 7 compounds associated with AS disease (Table 4) (Figure 8A).

**TABLE 4:** Compounds of HYQT medicated serum.

No.	Compound CID	Name	Formula	Molecular weight	RT [min]	mzCloud best match	mzVault best match
1*	6274	L‐Histidine	C6H9N3O2	155.06954	1.219	95.5	98.3
2*	5,375,048	Indole‐3‐acrylic acid	C11H9NO2	187.06318	7.396	94.7	94.3
3*	1123	Taurine	C2H7NO3S	125.0147	1.269	93.9	95.7
4*	288	DL‐Carnitine	C7H15NO3	161.10511	1.199	93	96.2
5*	5,280,644	9(Z),11(E)‐Conjugated linoleic acid	C18H32O2	280.23958	22.37	92.4	93.6
6*	69,421	Hexadecanamide	C16H33NO	255.25577	21.77	92.1	92.7
7*	7,045,767	Acetyl‐L‐carnitine	C9H17NO4	203.11557	1.382	90.2	98.8
8	461	Palmitoylcarnitine	C23 H45 N O4	399.3344	17.71	93	90.8
9	6426853	Hexanoylcarnitine	C13 H25 N O4	259.17839	9.227	93.3	98.6
10	11,487	Diphenylamine	C12 H11 N	169.08907	16.845	95.6	93.9
11	10,466	16‐Hydroxyhexadecanoic acid	C16 H32 O3	272.23497	22.119	95.1	95.9

*Note*: ‘*’ indicated the compounds associated with AS disease.

### 3.11 Molecular docking of HYQT medicated serum compounds with mTOR protein

Molecular docking was further performed to screen active components, which identified seven compounds associated with AS disease. Software Autodock vina 1.5.6 was used to obtain molecular docking, and (Table 5) summarized the results of the interaction energy. The poses of seven core compounds with mTOR protein were shown in (Figure 8B).

**TABLE 5:** The interaction energy values of component with the ligand protein mTOR.

Component	Interaction energy value (Kcal/mol)
L‐histidine	−5.0
Indole‐3‐acrylic acid	−7.4
Taurine	−3.9
DL‐carnitine	−4.3
9(Z),11(E)‐conjugated linoleic acid	−5.5
Hexadecanamide	−5.7
Acetyl‐L‐carnitine	−4.4

### 3.12 Mechanism of taurine‐the main component of HYQT

Taurine, a main component in HYQT. Previous studies have found that taurine can regulate autophagy and regulate mTOR protein expression.^30^ In this study, the effect of taurine on lipid accumulation in RAW 264.7 cells was detected. Furthermore, WB analysis was used to verify the regulation of taurine on lipophagy and mTORC1/TFEB signalling pathway.

**FIGURE 9:**
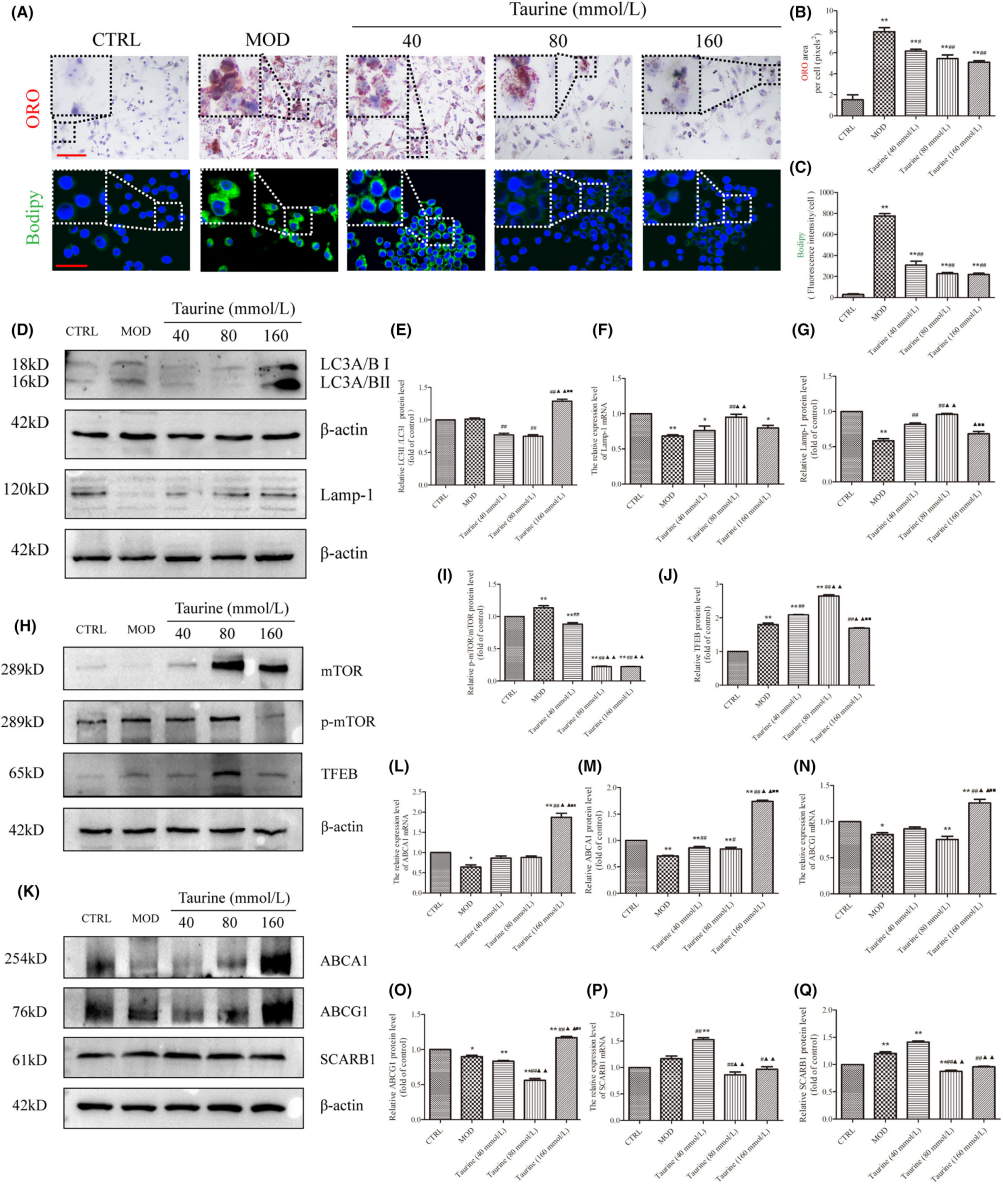
Taurine regulates lipid accumulation and mTOR/TFEB/ABCA1‐SCARB1 signal axis. (A) Taurine inhibits lipid droplet accumulation (BODIPY Scale bar: 25 μm, ORO Scale bar: 100 μm). (B,C) Normalized ratios of BODIPY and ORO staining. (D–G) Taurine promotes LC3II/I and Lamp1 proteins. (H–J) Taurine regulates mTOR/TFEB signalling pathway. (K) Taurine regulates cholesterol efflux proteins. (L,M) ABCA1 mRNA and protein, (N,O) ABCG1 mRNA and protein, (P‐Q) SCARB1 mRNA and protein. qRT‐PCR and WB data were presented as the mean ± SD (*n* = 3). *P < 0.05, **P < 0.01 versus CTRL. ^#^ *P* < 0.05, ^##^ *P* < 0.01 versus MOD, ^▲^ *P* < 0.05, ^▲▲^ *P* < 0.01 versus taurine (40 mmol/L). ^■^ *P* < 0.05, ^■■^ *P* < 0.01 versus taurine (80 mmol/L).

The ORO and BODIPY staining (Figure 9A) results showed that BODIPY fluorescence intensity and area of ORO in single cells were significantly increased in MOD and taurine group compared with CTRL group. Compared with MOD group, BODIPY fluorescence intensity and the area of ORO in taurine 40, 80 and 160 mmol/L groups decreased differently (Figure 9B,C).

The WB results showed that LC3II/I level in taurine (160 mmol/L) was increased significantly compared with MOD group, and increased significantly in taurine (160 mmol/L) group compared with taurine (40 and 80 mmol/L) groups. Lamp‐1 mRNA and protein level in MOD group was decreased significantly compared with CTRL group (*P* < 0.01). Lamp‐1 mRNA increased in taurine (40, 80 and 160 mmol/L) groups, and taurine 80 mmol/L group was increased significantly compared with taurine 40 mmol/L group. The protein of Lamp‐1 showed the same results with mRNA (Figure 9D–G). The p‐mTOR/mTOR protein level in MOD group was increased compared with CTRL group. Compared with MOD group, p‐mTOR/mTOR protein level was decreased after treated with taurine. The TFEB protein level increased in MOD and taurine (40, 80, and 160 mmol/L) groups compared with CTRL group. While, compared with MOD group the TFEB protein level increased in taurine (40, 80 mmol/L) groups. Taurine 80 mmol/L group was increased significantly compared with 40 mmol/L group. TFEB protein level in taurine (160 mmol/L) group decreased significantly compared with MOD, taurine (40, 80 mmol/L) groups (Figure 9H–J).

Cholesterol efflux proteins ABCA1, ABCG1, SCREB1 changed after treated with taurine. Compared with CTRL group, the ABCA1 mRNA level decreased. Compared with CTRL, MOD, taurine (40, 80 mmol/L) groups, ABCA1 mRNA level increased in taurine (160 mmol/L) group. Compared with CTRL group, ABCA1 protein level decreased in MOD, taurine (40 and 80 mmol/L) groups. Compared with MOD group, the ABCA1 protein level in taurine (40, 80, and 160 mmol/L) groups increased significantly. Compared with taurine (80 mmol/L) group, the ABCA1 protein level in taurine (160 mmol/L) group increased significantly. Compared with the CTRL group, ABCG1 mRNA in MOD group was decreased; compared with MOD group, taurine (40, 80 mmol/L) groups were increased. ABCG1 protein level in MOD, taurine (40, 80 mmol/L) groups decreased compared with the CTRL group. ABCG1 protein level was increased in taurine (160 mmol/L) group compared with the MOD, taurine (40, 80 mmol/L) groups. SCARB1 mRNA level increased in taurine (40 mmol/L) group compared with the CTRL and MOD groups. Taurine (80 and 160 mmol/L) groups decreased significantly compared with taurine (40 mmol/L) group. SCARB1 protein level increased in MOD and taurine (40 mmol/L) groups, compared with CTRL group. Compared with taurine (40 mmol/L) group, the SCARB1 protein level decreased in taurine (40, 80 mmol/L) groups (Figure 9K–Q).

## 4 DISCUSSION

Atherosclerosis is a chronic disease characterized by abnormal lipid metabolism, macrophage foaming and plaque deposition. The formation of macrophage‐derived foam cells plays a crucial role in the pathogenesis of atherosclerosis.^31^ Recent studies have shown that lipophagy, which is the activation of autophagy, is involved in the breakdown of lipid droplets for the maintenance of lipid homeostasis.^32^ This process allows the LDs to be transported to lysosomes for degradation, thereby inhibiting the formation of foam cells.

Lipophagy, which refers to the autophagic removal of lipids, plays a significant role in macrophage cholesterol efflux and is closely associated with atherosclerosis.^33^ The induction of macrophage autophagy–lysosomal biogenesis enhances lipophagy and facilitates the transportation of cholesteryl esters, resulting in the suppression of inflammatory response and improvement of atherosclerosis.^34^ Jeong SJ's study further confirmed the crucial role of lipophagy in controlling atherosclerosis in vivo.^33^

Besides, the phenomenon of lipophagy extends beyond atherosclerotic disease. In their study, Marschallinger et al. discovered that microglia, which accumulate lipid droplets, also possess a significant number of lysosomes. Moreover, these lysosomes tend to accumulate in close proximity to the lipid droplets.^35^

Yang YP discovered that hyperhomocysteinaemia inhibits autophagosome formation in human THP‐1 macrophages through the AMPK‐mTOR‐TFEB signalling pathway.^36^ When nutrients are present, TFEB co‐localizes with the MTOR complex on the lysosomal membrane, and phosphorylation of TFEB by MTOR inhibits TFEB activation.^37^ Pi H et al found that modulation of the SCD1/TFEB mediated lipophagy machinery may offer novel therapeutic approaches for the treatment of atherosclerosis.^38^

The mTOR/TFEB signalling pathway plays a crucial role in the inhibition of lipophagy by ox‐LDL. HYQT regulates the degradation of lipid droplets through mTOR/TFEB and effectively suppresses foam cell formation. This study demonstrates that HYQT modulates lipophagy via the mTOR/TFEB signalling pathway, rather than the mTOR/4EBP1/P70S6K signalling pathway.

In vivo, this study provides evidence that HYQT inhibits the development of atherosclerosis in ApoE^−/−^ mice and reduces lipid deposition in atherosclerotic plaques. The study also confirms the decrease in lipid droplet accumulation in macrophages. Transmission electron microscopy was used to observe the aortic plaque, and the study identified the presence of macrophages as well as the formation of lysosomes and autophagosomes within them (Figure 1). To gain further insights into the mechanism, in vitro experiments were conducted. At first, the formation of ox‐LDL‐induced foam cells at different time periods can lead to changes in cell cholesterol content. In this study, the choice of 48 hours as the time period is based on the following reasons: Previous studies have demonstrated that short‐term stimulation with ox‐LDL (6 h, 12 h and 24 h) enhances lipophagy levels due to compensatory feedback, thereby maintaining lipid metabolism homeostasis. However, prolonged treatment (48 h) with ox‐LDL affects the level of lipid accumulation in macrophages. When macrophages were stimulated with ox‐LDL for 48 h, there was a significant increase in the levels of CE. In this study, foam cell model was constructed using 50 μg/ mL ox‐LDL for 48 h (Figure 2A–C). Next, the optimal concentrations and times of HYQT‐medicated serum was screened. Ultimately, the results found that cells were incubated with 10% medicated serum for 24 hours for optimal cell activity (Figure 2D).

Chloroquine, an autophagy lysosome inhibitor, was found to promote lipid accumulation in macrophages according to the results of this study. The study involved treating macrophages with CQ (50 μM) for different time intervals (0, 2, 6, 12, 24 and 48 h) and observing changes in lamp‐1 and LC3II/I proteins. The results indicate that autophagic flow was inhibited after treating RAW 264.7 cells with CQ (50 μM) for 48 h. Hence, it can be hypothesized that the distinct time course of chloroquine's action on macrophages impacts the formation of foam cells. This effect may be attributed to the extended inhibition of lysosomes, which ultimately inhibits the degradation of lipid droplets (Figure 2E–K).

Rapamycin, an autophagy agonist and mTOR inhibitor, has been applied in this study to assess the impact of HYQT on autophagy levels. This effect may be attributed to the silencing of mTOR and holds significant importance for preliminary evaluation. First, to investigate the impact of HYQT on lipid accumulation, ORO staining and BODIPY staining were performed. The results showed that HYQT has an inhibitory effect on lipid droplet deposition. However, medicated control serum has no effect on lipid droplet accumulation. Changes in lipid droplets accumulation with changing autophagy flux levels, which was different in the CQ and RAPA groups (Figure 2L–N). Similar study has demonstrated that the lack of autophagy reduces the degradation of lipid droplets.^39^

Then, the formation of autophagosomes was detected via transmission electron microscopy (Figure 3A). HYQT, CQ and RAPA affected the formation of autophagosomes. Immunofluorescence and fluorescent probe experiments showed that HYQT reduced the fluorescence intensity of lipid droplets, increased the per cell fluorescence intensity of the lysosomal probe and enhanced the co‐localization of lipid droplets with lysosomes and LC3b (Figure 3B–E). Autophagy genes and proteins assay have similar results to the above experiments (Figure 4). This part of the experimental results was consistent with the previous experiments, which demonstrated that HYQT could regulate the degradation of lipid droplets through the lysosomal pathway.

As we know, the causes of lipid accumulation in macrophages mainly include decreased cholesterol efflux. Therefore, it is important to investigate the mechanism of HYQT inhibits foam cell formation and promotes cholesterol efflux. The expression levels of mTOR, p‐mTOR, TFEB, p‐4EBP1, 4EBP1, p‐P70S6K, P70S6K, ABCA1, ABCG1 and SCARB1 proteins in RAW264.7 cells were detected via WB analysis (Figure 5A). In this study, HYQT regulates mTOR/TFEB signalling pathway, promotes TFEB level into the nucleus. HYQT increased the expression of ABCA1, ABCG1 and SCARB1 proteins level. Conclusion of this part was HYQT regulates lipophagy may not depend on mTOR/4EBP1/P70S6K signalling pathway (Figure 5B–M). Finally, MHY1485 was used to explore the correlation between the mTOR/TFEB signalling pathway with lipophagy and cholesterol efflux. The result found that MHY1485 reversed the effect of HYQT on lipid droplets accumulation (Figure 6A–C). MHY1485 inhibited the co‐localization of BODIPY with lysosomes and LC3b (Figure 6D–G). Specifically, this study observed that the MHY1485 reduced the ratio of cholesterol efflux compared (Figure 6H,I), which indicated that HYQT enhances cholesterol efflux may via regulating mTOR signalling pathway. Then, TFEB, LC3I/II, Beclin, p62, ABCA1, ABCG1 and SCARB1 proteins were detected in this study. Interestingly, a decrease in ABCA1 and SCARB1 proteins expression were observed when mTOR was activated in the MHY1485 group (Figure 7H–N). ABCG1 protein showed no change after treatment with MHY1485. The results proved that MHY1485 reversed the effect of HYQT on ABCA1 and SCARB1. Additionally, DMSO, which served as the solvent for MHY1485 and CQ, had no effect on the results. ABCA1 and SCARB1 may the downstream molecule of mTOR/TFEB in this model.

The chemical composition of the medicated serum was detected. The findings indicated that the serum contains 7 beneficial components that have shown effectiveness in treating AS (Figure 8). Hasegawa S demonstrated that L‐histidine could anti‐inflammatory in human coronary arterial endothelial cells.^40^ Additionally, microbial metabolites like indole‐3‐acrylic acid have been shown to potentially reduce the progression of atherosclerosis.^41^ Qaradakhi T discovered that taurine has anti‐inflammatory effects, which could treat diabetes and prevent cardiovascular disease.^42^ In addition, Singh P emphasized the taurine is linked to aging and cellular health, which may derive from lipophagy/cholesterol efflux.^43^According to a report published in the Lancet, patients with Type IV hyperlipoproteinaemia experienced a significant reduction in serum triglycerides when treated with DL‐carnitine at a dose of 900 mg/d, studied by Maebashi M.^44^ 9(Z),11(E)‐Conjugated linoleic acid has been shown to promote fat metabolism, while hexadecanamide has been found to reduce inflammation.^45^ Additionally, linoleic acid has been identified as a potential anti‐obesogenic agent.^46^, ^47^ Wang S discovered that acetyl‐L‐carnitine has the potential to inhibit the expression of inflammatory factors and promote antioxidation. This mechanism effectively suppresses the development of atherosclerosis in AS rats by regulating blood lipids in the myocardium.^48^To investigate the interaction between mTOR protein and a combination of seven compounds, molecular docking technique was employed to determine the free binding energy. The findings revealed that these compounds exhibit low energy when binding with mTOR protein, suggesting that they could potentially serve as the key compounds in HYQT for enhancing lipid phagocytosis. (Figure 8B).

Based on previous research results, taurine was used to treat RAW264.7 cells for this study. It was found that taurine is the key compound in HYQT, which regulates the mTOR/TFEB signalling pathway.

This study consistent with the research conducted by Xiaolong L, which demonstrated that lipophagy flux can enhance cholesterol efflux and decrease cholesterol accumulation in foam cells.^49^ Similar studies have found that attenuated lipophagy may lead to lipid accumulation in ox‐LDL‐treated endothelial cells (ECs).^50^ Kobayashi T discovered that in obese mice and hepatocytes, AMPK enhanced the movement of TFEB into the nucleus. Inhibition of AMPK resulted in TFEB nuclear localization, inhibited liver autophagy, and contributed to the development of hepatic steatosis.^51^ Additionally, Evans TD found that the decrease of SQSTM1/p62 protein was observed through the construction of TFEB overexpression in a mice model.^52^ In a study by Wang YT, the ability of TFEB to regulate autophagy was examined, and it was found to block pathological vascular remodelling.^53^

In summary, the key to treating atherosclerosis with HYQT depend on inhibiting foam cell formation. This can be achieved by promoting the lipophagy of macrophage, which in turn promotes cholesterol efflux. The mechanism of action may rely on the mTOR/TFEB/ABCA1‐SCARB1 signal axis. Additionally, this study highlights the potential role of taurine as a crucial compound in regulating this mechanism of HYQT after metabolism in vivo.

## 5 STRENGTHS AND LIMITATIONS

This study demonstrated that HYQT has the ability to inhibit the formation of macrophage‐derived foam cells and the accumulation of lipids. The results found that HYQT improves lipophagy by regulating the mTORC1/TFEB/ABCA1‐SCARB1 signal axis. Moreover, the mTORC1/TFEB signalling pathway regulates ABCA1. However, the underlying molecular interaction mechanism of this phenomenon requires further elucidation. It could potentially involve protein–protein interactions, transcription factors, RNA modification, non‐coding RNA interference and other factors. Future studies will go further in revealing the mechanisms.

## 6 CONCLUSIONS

This study reveals how HYQT affects cholesterol efflux from macrophage‐derived foam cells. HYQT regulates the mTORC1/TFEB/ABCA1‐SCARB1 signal axis to improve cholesterol efflux. These findings suggest that HYQT has the potential to be used as a therapeutic option for patients with lipid metabolism disorders to slow the progression of atherosclerosis.

## AUTHOR CONTRIBUTIONS

**Yue Li:** Methodology (lead); project administration (lead); writing – original draft (lead); writing – review and editing (lead). **Jiaxiang Pan:** Conceptualization (equal); data curation (equal); methodology (equal); project administration (equal); writing – original draft (lead); writing – review and editing (lead). **J. J. Jiajia Yu:** Methodology (equal); resources (equal); writing – review and editing (equal). **Xize Wu:** Investigation (equal); methodology (equal); writing – review and editing (equal). **Guanlin Yang:** Funding acquisition (equal). **Xue Pan:** Visualization (equal). **Guoyuan Sui:** Supervision (equal). **Mingyang Wang:** Investigation (equal); methodology (equal). **Meijia Cheng:** Resources (equal); software (equal). **Shu Zhu:** Validation (equal). **He Tai:** Conceptualization (equal); data curation (equal). **Honghe Xiao:** Validation (equal). **Lili Xu:** Data curation (equal). **Jin Wu:** Methodology (equal). **Yongju Yang:** Resources (equal). **Jing Tang:** Validation (equal). **Lihong Gong:** Funding acquisition (equal); project administration (lead); supervision (lead). **Lianqun Jia:** Funding acquisition (equal); project administration (lead); supervision (lead). **Dongyu Min:** Formal analysis (equal); project administration (lead); supervision (lead).

## FUNDING INFORMATION

This work was supported by National Natural Science Foundation of China in 2021 (82074145), National Natural Science Foundation of China in 2019(81974548),Science and Technology Research Project of Education Department of Liaoning Province (No.L202048), Youth Project of Basic Scientific Research Project of Education Department of Liaoning Province (No.LJKQZ2021064), The ‘Seedling Raising Project’ of The Affiliated Hospital of Liaoning University of Chinese Medicine in 2021(No.YM202109), and Science and Technology Research of Education Department of Liaoning Province (No.L201722),Open Fund of Key Laboratory of Theory and Application of Viscera‐ manifestation theory in Traditional Chinese Medicine, Ministry of Education, Liaoning University of TCM (ZYZX1907).

## CONFLICT OF INTEREST STATEMENT

The author reports no conflicts of interest in this work.

## Data Availability

The data that support the findings of this study are available on request from the corresponding author. The data are not publicly available due to privacy or ethical restrictions.
